# A parapoxviral virion protein targets the retinoblastoma protein to inhibit NF-κB signaling

**DOI:** 10.1371/journal.ppat.1006779

**Published:** 2017-12-15

**Authors:** Ponnuraj Nagendraprabhu, Sushil Khatiwada, Sabal Chaulagain, Gustavo Delhon, Daniel L. Rock

**Affiliations:** 1 Department of Pathobiology, College of Veterinary Medicine, University of Illinois at Urbana- Champaign, Urbana, IL, United States of America; 2 School of Veterinary and Biomedical Sciences, Center for Virology, University of Nebraska-Lincoln, Lincoln, NE, United States of America; University of Utah, UNITED STATES

## Abstract

Poxviruses have evolved multiple strategies to subvert signaling by Nuclear Factor κB (NF-κB), a crucial regulator of host innate immune responses. Here, we describe an orf virus (ORFV) virion-associated protein, ORFV119, which inhibits NF-κB signaling very early in infection (≤ 30 min post infection). ORFV119 NF-κB inhibitory activity was found unimpaired upon translation inhibition, suggesting that virion ORFV119 alone is responsible for early interference in signaling. A C-terminal LxCxE motif in ORFV119 enabled the protein to interact with the retinoblastoma protein (pRb) a multifunctional protein best known for its tumor suppressor activity. Notably, experiments using a recombinant virus containing an ORFV119 mutation which abrogates its interaction with pRb together with experiments performed in cells lacking or with reduced pRb levels indicate that ORFV119 mediated inhibition of NF-κB signaling is largely pRb dependent. ORFV119 was shown to inhibit IKK complex activation early in infection. Consistent with IKK inhibition, ORFV119 also interacted with TNF receptor associated factor 2 (TRAF2), an adaptor protein recruited to signaling complexes upstream of IKK in infected cells. ORFV119-TRAF2 interaction was enhanced in the presence of pRb, suggesting that ORFV119-pRb complex is required for efficient interaction with TRAF2. Additionally, transient expression of ORFV119 in uninfected cells was sufficient to inhibit TNFα-induced IKK activation and NF-κB signaling, indicating that no other viral proteins are required for the effect. Infection of sheep with ORFV lacking the ORFV119 gene led to attenuated disease phenotype, indicating that ORFV119 contributes to virulence in the natural host. ORFV119 represents the first poxviral protein to interfere with NF-κB signaling through interaction with pRb.

## Author summary

Poxviruses have evolved multiple strategies to subvert signaling by NF-κB, a crucial regulator of host innate immune responses. Viruses often encode multiple inhibitory proteins, which largely target cytoplasmic activation events of NF-κB signaling. The retinoblastoma protein (pRb), a multifunctional protein best known for its tumor suppressor activity, has been suggested to affect NF-κB signaling during virus infection however, viral effectors and mechanisms of actions are unknown. Here, we identified a virion-associated orf virus NF-κB inhibitory protein, ORFV119, which interacts with pRb. ORFV119 was shown to inhibit IKK complex activation in a pRb-dependent manner early in infection. Results show that ORFV119 interacted with both pRb and TRAF2 and that a ORFV119-pRb complex likely is required for efficient interaction with TRAF2 and inhibition of NF-κB signaling. ORFV119 represents the first poxviral protein to interfere with NF-κB signaling through interaction with pRb.

## Introduction

Orf virus (ORFV), the type member of the genus Parapoxvirus of the Poxviridae, is the causative agent of orf or contagious ecthyma, a ubiquitous disease of sheep and goats characterized by proliferative lesions affecting muco-cutaneous tissues of the mouth and muzzle [1,2]. Orf is a zoonotic disease affecting humans in close contact with infected animals [3–5].

Like other parapoxviruses (PPV), ORFV is highly epitheliotropic, and keratinocytes and their ontogenetically related counterparts in the oral mucosa are the most important if not the only cell type to support ORFV replication *in vivo* [6]. In addition to producing the essential protective stratum corneum of the epidermis, keratinocytes function as immune sentinels and instigators of inflammatory responses in the skin, representing a specialized branch of the innate immune system. Keratinocytes are well equipped for pathogen sensing as they express a broad spectrum of pattern recognition receptors (PRRs), including surface and endosomal toll-like receptors (TLRs), NOD-like receptors (NLRs), and retinoic acid-inducible gene (RIG-I)-like receptors, and rapidly respond to cell injury and infection by releasing critical pro-inflammatory chemokines and cytokines such as tumor necrosis factor α (TNFα) and interleukin 1 (IL-1) [7,8]. Engagement of these receptors initiates downstream pro-inflammatory cascades, including the NF-κB signaling pathway, which mediates innate immune responses and contributes to skin homeostasis by regulating keratinocyte proliferation and differentiation [9].

The NF-κB family of transcription factors consists of five members, NF-κB-p65 (RelA), RelB, c-Rel, NF-κB-p50/p105, and NF-κB-p52/p100, which contain an N-terminal Rel homology domain (RHD) responsible for homo- and heterodimerization and for sequence specific DNA binding [10]. In unstimulated cells, NF-κB dimers are sequestered in the cytoplasm through binding to the inhibitor kappa-B alpha (IκBα). Following cell stimulation, IKK complex-mediated phosphorylation of IκBα results in proteasomal degradation of IκB and nuclear translocation of p65/p50 dimers, which bind κB-responsive DNA elements, interact with transcription co-regulators, and activate or repress gene expression [11,12]. The critical IKK complex consists of two kinases, IKKα and IKKβ, and the regulatory subunit IKKγ/NF-κB essential modulator (NEMO) [13,14]. Various stimuli, including those initiated by proinflammatory cytokines TNFα and IL-1, lead to IKK activation. Engagement of the TNF receptor 1 (TNF-R1) results in sequential recruitment of TRADD (TNF-R1-associated death domain), TRAF2 (TNF receptor-associated factor 2) and RIP1 (Receptor-interacting protein 1) [15]. Multiple ubiquitination events on RIP1 and NEMO bring the TAK1 (TGF-β activated kinase 1) complex close to the IKK complex. TAK1-mediated IKKβ phosphorylation and IKKβ auto-phosphorylation activate IKKβ, which then phosphorylates IκBα [16]. Engagement of the IL-1 receptor, on the other hand, results in recruitment of IRAK1 (IL-1 receptor–associated kinase) and activation of TRAF6 (TNF receptor-associated factor 6), which then ubiquitinates and activates TAK1 resulting in IKK activation [17,18].

Many viruses with dissimilar life styles are known to interfere with the NF-κB pathway. In particular, poxviruses have evolved multiple strategies to counteract NF-κB function, indicating that inhibition of NF-κB-mediated transcription is important for successful infection of the host. This is not surprising as poxvirus infections are sensed by NF-κB-activating PRRs such as endosomal TLRs, RIG-I-like receptors, and the inflammasome [19]. General features of poxviral NF-κB inhibitors include, 1- individual viruses encode for multiple inhibitors, with vaccinia virus (VACV) encoding at least twelve [20]. While orthologs of some NF-κB inhibitors are found in viruses belonging to multiple poxvirus genera (e.g. VACV A52R, VACV E3L), others are restricted to a particular genus (e.g. VACV A46R and VACV B14R in Orthopoxvirus) or even selected viruses within a genus (PPV ORFV002) [21–29]; 2- in contrast to other classes of poxviral immunomodulators, poxviral NF-κB inhibitors have no or little resemblance to host proteins; 3- although inhibitors target extracellular, membrane, cytosolic, or nuclear events of NF-κB regulation, most inhibitors directly target NF-κB subunits or the proximal IKKs; 4- despite the multiplicity of inhibitors, there seems to be low or no redundancy as judged by the effect of individual gene deletions on viral pathogenesis; 5- with a few exceptions (myxoma virus MYXV150, cowpoxvirus CPXV006) no single gene-deletion rendered complete virus attenuation [30–34]; 6- most inhibitors are expressed early after virus entry into cells.

Apart from VACV E3L (ORFV020), PPV lack homologues of poxviral NF-κB inhibitors present in other poxviral genera, suggesting that PPV have evolved novel mechanisms to counteract the NF-κB signaling pathway. Recently, we have described four NF-κB inhibitors encoded by ORFV, ORFV024, ORFV002, ORFV121 and ORFV073 [35–38]. ORFV024 was shown to inhibit phosphorylation of IKK kinases, thus preventing activation of IKK complex. ORFV121, a virulence determinant, was shown to bind to- and inhibit phosphorylation and nuclear translocation of NF-κB-p65 [37]. ORFV002 binds NF-κB-p65 and reduces its acetylation by co-activator p300 thus inhibiting transactivation [36]. Decreased NF-κB-p65 acetylation is a consequence of ORFV002 interfering with NF-κB-p65 phosphorylation by mitogen- and stress-activated protein kinase 1 (MSK1) [39]. ORFV073 inhibits NF-κB signaling by preventing activation of IKK complex through interaction with NEMO, the regulatory subunit of the IKK complex [38].

The retinoblastoma tumor suppressor, pRb, is a multifunctional, predominantly nuclear protein encoded by the RB1 gene. pRb affects cell cycle, differentiation and metabolism, genome stability and apoptosis, mostly but not exclusively through transcription regulation [40]. Central to pRb function is its ability to nucleate complexes containing multiple interacting partners, thus participating in various regulatory circuits. Viruses have evolved functions to modulate those pathways by targeting pRb to their advantage. For example, adenovirus (Ad) protein E1A interaction with pRb and other factors represses select host genes to promote productive virus infection [41]. The human cytomegalovirus pp71 tegument protein and the human papillomavirus E7 oncoprotein bind to pRb and induce its degradation thus driving cells to a cell cycle stage that potentially favors efficient replication of viral genomes [42,43]. pRb has been shown to affect the regulation of the NF-κB pathway following TNFα signaling or vesicular stomatitis virus infection, although mechanisms involved are not yet defined [44,45]. Many viral and cellular pRb-interacting proteins contain the motif LxCxE (where x means any amino acid) that binds to the LxCxE cleft of the pRb pocket domain [46]. Select pRb-binding proteins that interact through LxCxE with pRb also bind p107 and p130, the other two members of the retinoblastoma family of proteins.

Here we show that ORFV119, a LxCxE motif-containing virion protein unique to PPV, interferes with NF-κB signaling in a pRb-dependent manner early in infection by inhibiting IKK complex activation.

## Materials and methods

### Cells and viruses

Primary ovine fetal turbinate (OFTu) cells were obtained from Howard D. Lehmkuhl (USDA) and were maintained in minimal essential medium (MEM) supplemented with 10% fetal bovine serum (FBS) (Atlanta Biologicals, Flowery Branch, GA), 2 mM L-glutamine, gentamicin (50 μg/ml), penicillin (100 IU/ml), and streptomycin (100 μg/ml). Human osteosarcoma cells (Saos-2, provided by Timothy M. Fan, UIUC), Human embryonic kidney (HEK 293T) and cervical cancer (HeLa) cells (obtained from American Type Culture Collection), were cultured in Dulbecco's modified essential medium (DMEM) supplemented as above. Cells were incubated at 37°C with 5% CO2. ORFV strain OV-IA82 [47] was used as a parental virus to construct ORFV119 gene deletion virus OV-IA82-Δ119 and for experiments involving wild type virus infection. OV-IA82-Δ119 was used as parental virus to construct revertant virus OV-IA82-RV119^Flag^, and OV-IA82-RV119^LxGxE-Flag^, a revertant virus carrying a CxG substitution in ORFV119 LxCxE motif.

### pRb knockdown in OFTu cells (OFTu^Rb-^)

To knockdown pRb expression in OFTu cells, siRNA directed against ovine RB1 was used. A pool of three RB1 sense (S) and anti-sense (AS) siRNA with following sequences 1-RB1-(S): CTACCTATAGCAGAAGTAT, RB1-(AS): ATACTTCTGCTATAGGTAG; 2-RB1-(S): GCTTATATATTTGACACAA, RB1-(AS): TTGTGTCAAATATATAAGC; 3-RB1-(S): CTCAGATTCACCTTTATTT, RB1-(AS): AAATAAAGGTGAATCTGAG (Custom Oligos: Sigma Aldrich) were used. Pooled RB1 siRNA (15 nM of each siRNA) were transfected in OFTu cells (30,000–70,000/well) using MISSION transfection reagent (Sigma Aldrich, Cat # S1452) following the manufactures’ protocol. At 48 h post transfection, pRb knockdown was examined by SDS-PAGE, using antibody against pRb (abcam, Cat # ab85607). One MISSION siRNA Universal Negative Control (SIC001, sigma aldrich) was included in all experiments.

To obtain OV-IA82^Rb-^ or OV-IA82-RV119^LxGxE-Flag-Rb-^ virus stocks (OV-IA82 and OV-IA82-RV119^LxGxE-Flag^ viruses propagated in cells with reduced pRb levels, respectively), OFTu cells with reduced pRb levels (OFTu^Rb-^ cells) were infected with OV-IA82 or OV-IA82-RV119^LxGxE-Flag^ virus and supernatants from infected cultures were collected at 24 h p.i and used for NF-κB-p65 nuclear translocation assays.

### Plasmids

To construct expression plasmids pORFV119^Flag^ and pORFV119^LxGxE-Flag^, the *ORFV119* or *ORFV119*^*LxGxE*^ coding sequences were PCR-amplified from the OV-IA82 genome and a plasmid containing synthetic *ORFV119*^*LxGxE*^ sequence (Genscript), respectively with primers 119-3xFlag-FW (*EcoRI*): 5’-TAAGGCCTCTGAATTCAATGGACTCTCGTAGGCTC GCTCTT-3’; 119-3xFlag-RV (*BamHI*): 5’-CAGAATACGTGGATCCTCAATCGCTGTCG CTGTCGCCGAG-3’ and cloned into p3xFlag-CMV-10 vector (pFlag) (Clontech, Mountain View, CA). Similarly, to obtain a ORFV119-green fluorescence protein (ORFV119^GFP^) expression vector, *ORFV119* sequence was PCR amplified from OV-IA82 genome with primers 119-GFP-FW (*EcoRI*): 5’- TAAGGCCTCTGAATTCATGGACTCTCGTAGGCTCGCTCTT-3’ and 119-GFP-RV (*BamHI*): 5’- CAGAATACGTGGATCCAGATCGCTGTCGCTGTCGCCGA GCG-3’, and cloned into the vector pEGFP-N1 (Clontech, Mountain View, CA). DNA sequencing of plasmids confirmed the identity and integrity of the constructs.

### Construction of *ORFV119* gene deletion mutant and revertant viruses

To generate gene deletion mutant virus OV-IA82-Δ119, a recombination cassette (pΔ119-RFP) containing Vaccinia virus 7.5 promoter (VV7.5)-driven Red Fluorescent Protein (*RFP*) gene flanked by 528 bp sequences representing *ORFV119* left and right flanking regions was synthesized and cloned in pUC57 vector (Genscript, Piscataway, NJ). Similarly, to generate revertant viruses OV-IA82-RV119^Flag^ and OV-IA82-RV119^LxGxE-Flag^, recombination cassettes pRV119^Flag-GFP^ and pRV119^LxGxE-Flag-GFP^ were synthesized containing N-terminally tagged ORFV119 or ORFV119^LxGxE^ sequences, respectively, and a GFP reporter gene, all flanked by approximately 600 bp of homologous sequence on either side to mediate recombination (Genscript, Piscataway, NJ).

To obtain OV-IA82-Δ119, OFTu cells were infected with OV-IA82 (MOI, 1) and transfected with recombination plasmid pΔ119-RFP. To obtain OV-IA82-RV119^Flag^ and OV-IA82-RV119^LxGxE-Flag^, OFTu cells were infected with OV-IA82-Δ119 (MOI, 1) and transfected with recombination plasmids pRV119^Flag-GFP^ or pRV119^LxGxE-Flag-GFP^. Recombinant viruses were isolated by limiting dilution and plaque assay using fluorescence microscopy as previously described [36]. Identity and integrity of DNA sequences in purified viruses was confirmed by PCR and DNA sequencing.

### Virus purification and virion protein characterization

Semi-purified viruses were used in infection experiments. OFTu cells infected with OV-IA82, OV-IA82-RV119^Flag^, OV-IA82-Δ119, or OV-IA82-RV119^LxGxE-Flag^ (MOI, 0.1) were disrupted by three cycles of freeze and thaw at three days p.i. Cellular debris were removed by centrifugation at 1500 rpm for 5 min, and supernatants pelleted by ultracentrifugation at 25,000 rpm for 1 h. Pellets were resuspended in MEM and aliquots frozen at -80°C. Viral titers were obtained by the Spearman-Karber’s 50% tissue culture infectious dose (TCID_50_) method.

Extracellular enveloped virus (EEV) and intracellular mature virus (IMV) were purified using double sucrose gradient purification protocol with modifications [48]. OFTu cells infected with OV-IA82, OV-IA82-Δ119, OV-IA82-RV119^LxGxE-Flag^ or OV-IA82-RV119^Flag^ (MOI, 0.1) were harvested at three days p.i. and supernatant (EEV) and cellular (IMV) fractions collected following centrifugation at 1,500 rpm for 5 min. Virus in supernatants was pelleted by ultra-centrifugation (25,000 rpm for 1 h), while cellular fractions were disrupted by three cycles of freeze and thaw and centrifuged to remove cell debris. Further purification steps were identical for EEV and IMV. Both preparations were sonicated, pelleted through a 36% sucrose cushion, and purified using double sucrose gradient centrifugation. Virus-containing bands were collected, and virus was recovered by centrifugation and resuspended in 10 mM TrisHcl. Whole cell extracts (60 μg) from OV-IA82-RV119^Flag^ infected OFTu cells, and purified EEV and IMV virion proteins (10 μg) were resolved by SDS-PAGE, blotted to nitrocellulose membranes and probed with primary antibodies against Flag (Genscript, Cat # A00187), ORFV structural protein ORFV086 or Glyceraldehyde-3-Phosphate Dehydrogenase (GAPDH) (sc-25778; Santa Cruz) overnight at 4°C [49]. Blots were incubated with appropriate HRP-labeled secondary antibodies (1:15,000) (anti-mouse, sc-2031 and anti-rabbit, sc-2004; Santa Cruz) for 1 h at room temperature (RT) and membranes were developed using chemiluminescent reagents (SuperSignal West Femto, Thermo Fischer Scientific).

To examine the effect of protein synthesis inhibitor cycloheximide (CHX) on expression of ORFV119 and control cellular protein p53. OFTu cells mock treated or treated with CHX (50 μg/ml) (Sigma-Aldrich, St. Louis, MO) for 30 min were infected with OV-IA82RV119^Flag^ in presence or absence of CHX and harvested at 30 min, 1 h, 1.5 h and 2 h p.i. Total protein extracts (50 μg) were resolved by SDS-PAGE, blotted and transferred to nitrocellulose membranes, probed with antibody against Flag, p53 (sc-6243; Santa Cruz) and GAPDH (sc-25778; Santa Cruz) as described above.

### ORFV119 protein expression and subcellular localization

To evaluate ORFV119 expression, OFTu cells were infected with OV-IA82 or OV-IA82-RV119^Flag^ (MOI, 10) for 2 h, 4 h, 6 h, 8 h, 12 h or 24 h p.i. Total protein extracts (50 μg) were resolved by SDS-PAGE, blotted and transferred to nitrocellulose membranes, and probed with primary antibody against Flag and GAPDH. Blots were then incubated with appropriate HRP-labeled secondary antibodies and developed using chemiluminescent reagents.

To assess the subcellular localization of ORFV119, OFTu cells (1.5 x 10^5^ cells/well) were cultured in four-well chamber slides (ibidi, Martinsried, Germany) for 16 h and mock infected or infected with OV-IA82-Δ119 or OV-IA82-RV119^Flag^ (MOI, 10). Cells were fixed at 3 h, 6 h, 12 h, 16 h or 24 h p.i with 4% formaldehyde for 20 min, permeabilized with 0.2% Triton X 100 for 10 min, blocked with 1% bovine serum albumin (Sigma-Aldrich, St. Louis, MO) for 1 h, and incubated with primary antibody against Flag (Cat # A00187-200; Genscript) overnight at 4°C. Cells were then stained with Alexa fluor 594 labelled secondary antibody (Thermo Fisher Scientific, Cat # A-11005), counterstained with DAPI (2 μg/ml) for 10 min and visualized by confocal microscopy (A1, Nikon).

### Growth curves

To assess the effect of ORFV119 in virus replication, one-step growth curves were determined in OFTu cells infected with OV-IA82, OV-IA82-Δ119 or OV-IA82-RV119^Flag^ (MOI, 10). Virus yields were quantitated at 6 h, 12 h, 24 h, 36 h, and 48 h p.i. as described above.

### Real-time PCR

To investigate the effect of OV-IA82-Δ119 infection on expression of NF-κB regulated genes, OFTu cells were mock infected or infected with OV-IA82, OV-IA82-Δ119 or OV-IA82-RV119^Flag^ (MOI, 10) and harvested at 2 h p.i. RNA was extracted using RNeasy Mini Kit (QIAGEN, Cat # 74104) and reverse transcribed as previously described [35]. mRNA for select NF-κB regulated genes was quantified using ABI Real time PCR system (Applied Biosystems, Foster city, CA), Power SYBR Green PCR Master Mix (Cat # 4368708, Applied Bio) and the following primers, TNFα (Fwd: 5’-CCTTCAACAGGCCTCTGGTT-3’; Rev: 5’-GTGGGCTACCGGCTTGTTAT-3’) IL1β (Fwd 5’-AAATCCCTGGTGCTGG ATAG-3’; Rev: 5’-GTTGTCTCTTTCCTCTCCTTGT-3’) NF-κB1 (Fwd: 5’- CAGAGAGGA TTTCGTTTCCGT-3’; Rev: 5’-TGCAGATTTTGACCTGAGGGT-3’) IL36α (Fwd: 5’-ATGTCTTCACACCTTGGCAGT-3’; Rev: 5’-ATCGGGTGTACCCTGGATAA-3’) TLR2 (Fwd: 5’-TTGCTCCTGTGACTTCCTGTC-3’; Rev: 5’- GAGCGTCACAGCGGTAGC-3’). Data analysis was performed as previously described [35]. Experiments were conducted with biological triplicates and at least three technical replicates. Statistical analysis was performed by using Student’s t test.

### NF-κB-p65 nuclear translocation assay

The effect of ORFV119 in TNFα-induced activation of NF-κB was assessed by NF-κB-p65 nuclear translocation assay. HeLa cells transfected with control plasmid (pEGFP-N1) or a plasmid encoding ORFV119-GFP fusion protein (pORFV119^GFP^) were treated with TNFα (20ng/ml) at 12 h post transfection for 30 min, fixed, permeabilized and blocked as described above, incubated with primary antibody against NF-κB-p65 (Cell Signaling, Cat # 8242s) for 2 h, stained with Alexa fluor 594-labeled secondary antibody (Thermo Fisher Scientific, Cat # A-11005) for 1 h, counterstained with DAPI, and examined by confocal microscopy. Numbers of GFP expressing cells (approximately 300 cells/sample) exhibiting nuclear NF-κB-p65 staining were determined in randomly selected fields and results were shown as mean percentage of GFP/ORFV119^GFP^ expressing cells containing nuclear NF-κB-p65 over three independent experiments. Statistical analysis of data was performed by using the Student’s t test.

To examine the role of ORFV119 on NF-κB-p65 nuclear translocation during ORFV infection, OFTu cells were mock infected or infected with OV-IA82, OV-IA82-RV119^Flag^, OV-IA82-Δ119 or OV-IA82-RV119^LxGxE-Flag^ (MOI, 10). Cells were fixed at 30 min, 1 h, 2 h, 4 h, and 6 h p.i. and processed for NF-κB-p65 staining as described above. Cells (approximately 300/sample) were randomly selected and scored as mean percentage of cells containing nuclear NF-κB-p65 over three independent experiments. Statistical analysis of data was performed by using Student’s t test.

To investigate the effects of CHX on NF-κB-p65 nuclear translocation during ORFV infection, OFTu cells were pre-treated with CHX for 30 min, infected with OV-IA82 or OV-IA82-Δ119 in presence or absence of CHX, and fixed at 30 min or 1 h p.i. NF-κB-p65 nuclear translocation assay, scoring, quantification and analysis were performed as described above.

To investigate the effect of OV-IA82, OV-IA82^Rb-^, OV-IA82-RV119^LxGxE-Flag^ and OV-IA82-RV119^LxGxE-Flag-Rb-^ virus infection on NF-κB-p65 nuclear translocation, OFTu or OFTu^Rb-^ cells were infected with OV-IA82, OV-IA82^Rb-^, OV-IA82-RV119^LxGxE-Flag^ or OV-IA82-RV119^LxGxE-Flag-Rb-^ virus (MOI, 10). Cells were fixed at 1 h p.i. and NF-κB-p65 nuclear translocation assay was performed as described above. Cells (approximately 300/sample) were randomly selected and scored for mean percentage of cells containing nuclear NF-κB-p65 over two independent experiments. Statistical analysis of data was performed by using Student’s t test.

Additionally, to assess the effect of pRb on NF-κB-p65 nuclear translocation, Saos-2 cells, a pRb negative cell line, were mock infected or infected with OV-IA82 or OV-IA82-Δ119 (MOI, 50) and fixed at 1 h, 1.5 h, 2 h, 4 h, and 6 h p.i. NF-κB-p65 nuclear translocation assay, quantification and analysis were performed as described above.

### Effect of ORFV119 expression on NF-κB-p65 signaling

HeLa cells transfected with control plasmid (pFlag) or a plasmid encoding ORFV119-3xFlag fusion protein (pORFV119^Flag^) were treated with TNFα (20ng/ml), and harvested 10 and 20 min post treatment. OFTu cells mock infected or infected with OV-IA82, OV-IA82-RV119^Flag^, OV-IA82-Δ119 or OV-IA82-RV119^LxGxE-Flag^ (MOI, 10) were harvested at 30 min and 1 h p.i. Total protein extracts (50 μg) were resolved by SDS-PAGE, blotted and transferred to nitrocellulose membranes and probed with specific antibody against phospho-IKKα/β (Ser176/180) (Cat # 2697; Cell Signaling), phospho-IκBα (Ser32/36) (Cat # 9246; Cell Signaling), phospho-NF-κB-p65 (Ser536) (Cat # 3033; Cell Signaling), IKKα/β (sc-7607; Santa Cruz), IκBα (sc-371; Santa Cruz), NF-κB-p65 (sc-7151; Santa Cruz) and GAPDH (sc-25778; Santa Cruz) or Flag. Blots were processed as described above. Protein bands were quantified for densitometric analysis using ImageJ software, Version 1.6.0 (National Institute of Health, Bethesda, MD) and fold changes calculated. Statistical analysis was performed by using Student’s t test.

To investigate the effect of ORFV infection on NF-κB-p65 activation, OFTu cells were infected with OV-IA82 or OV-IA82-Δ119 (MOI, 10) and harvested at 30 min, 1 h, 2 h, 4 h, and 6 h p.i. Total protein extract (50 μg) were resolved by SDS-PAGE, blotted and transferred to nitrocellulose membranes, probed with phospho-NF-κB-p65 and NF-κB-p65 antibodies, and developed as described above.

### Luciferase assays

The effect of ORFV119 on poly(I:C), poly(A:T) or ORFV DNA induced NF-κB-mediated transcriptional activity was investigated using a NF-κB promoter luciferase assay. Firefly luciferase gene under the control of NF-κB promoter (pNF-κB-Luc) and with a plasmid encoding sea pansy (Renilla reniformis) luciferase under the control of herpesvirus TK promoter (pRL-TK) were used in studies. HeLa cells cultured in 12-well plates were co-transfected with the vectors pNF-κB-Luc (450 ng; Clontech, Mountain View, CA), and pRL-TK (50 ng; Promega, Madison, WI) and pFlag or pORFV119^Flag^. At 24 h after transfection, cells were induced with poly(I:C) (Amersham, Pittsburgh, PA) (500ng), poly(A:T) (Inviogen, San Diego, CA) (750ng) or ORFV DNA (1μg). ORFV DNA was extracted from OV-IA82 virus stock using QIAamp DNA Blood Mini Kit (Qiagen, Germantown, MD). Luciferase activities were determined at 20 h post- induction using the Dual Luciferase Reporter Assay (Promega) and a luminometer. Data were analyzed as previously described [35]. Statistical analysis of the data was performed by using Student's t test.

To investigate the effect of ORFV119 on E2F transcriptional activity in ORFV infected cells, OFTu cells were co-transfected with a pE2F-Luc (450 ng; Signosis, Santa Clara, CA) and pRL-TK (50 ng) plasmids. At 24 h post transfection, cells were mock infected or infected with OV-IA82, OV-IA82Δ119, or OV-IA82-RV119^LxGxE-Flag^ (MOI = 10). Firefly and sea pansy luciferase activities were measured at 1, 2, 4 and 6 h p.i. and expressed as relative fold changes in luciferase activity as described above.

### Co-immunoprecipitation

The interaction of ORFV119 or ORFV119^LxGxE^ with pRb and ORFV119 with TRAF2 was assessed in virus-infected cells by co-immunoprecipitation. OFTu cells infected with OV-IA82-RV119^Flag^ or OV-IA82-RV119^LxGxE-Flag^ (MOI, 10) or mock infected were harvested at 12 h p.i. Total protein extraction and co-immunoprecipitation was performed using nuclear complex Co-IP Kit (Active Motif, Carlsbad, CA) following the manufacturer’s instructions. For co-immunoprecipitation, total protein extracts were incubated with antibodies against Flag, TRAF2 and pRb in the high stringency buffer (IP High buffer [Cat # 101676], 300 mM NaCl [Cat # 101684], detergent [Cat # 101683], 1M DTT [Cat # 3483-12-3; sigma], Protease inhibitor cocktail [Cat # P8340; sigma]) overnight at 4°C, and then incubated with pre-washed 50 μl slurry of protein G agarose beads (Cat # 16–266; Millipore) at 4°C for 2 h. Beads were washed four times with high stringency buffer (described above) and bound proteins were eluted in Laemmli buffer. For immunoblot analysis, eluted proteins and control total protein cell lysates were resolved by SDS-PAGE, blotted and transferred to nitrocellulose membranes, probed with antibodies against Flag, TRAF2 and pRb and developed as described above. Light chain specific antibody against Rabbit IgG (Cat # ab99697; Abcam) was used for TRAF2 blots. A flag-expressing protein ORFV113^Flag^ was used as a control for specificity of ORFV119 and TRAF2 interaction.

The interaction of pRb with TRAF2 in the presence of pORFV119^Flag^ also was assessed by co-immunoprecipitation. Antibodies for co-immunoprecipitation included anti-pRb (Cat # 9309s, Cell Signaling), anti-TRAF2 (Cat # sc-876, Santa cruz) and anti-Flag. Co-immunoprecipitations of total proteins extracts were performed as described above. The interaction of ORFV119 or ORFV119^LxGxE^ with putative cellular binding partners was assessed by co-immunoprecipitation in pORFV119^Flag^ or pORFV119^LxGxE-Flag^ transfected HEK 293T or HeLa cells at 12 h post transfection. Antibodies for co-immunoprecipitation included pRb (Fig 3A, B-Cat # ab85607, abcam) (Fig 3E, F-Cat # 9309s, Cell Signaling), TRAF2 (Cat # sc-876, Santa cruz), TAK1 (Cat # sc-7126, Santa cruz), RIP1 (Cat # 3493, cell signaling), TRAF6 (sc-7221) or NEMO (sc-8330). Co-immunoprecipitations of total proteins extracts were performed as described above.

The interaction of ORFV119 with TRAF2 also was assessed in Saos-2 cells, which do not express pRb. Saos-2 cells transfected with control plasmid (pFlag) and pORFV119^Flag^ or co-transfected with pRb (Origene, Rockville, MD) were harvested at 12h post transfection. Co-immunoprecipitations of total proteins extracts were performed as described above. Antibodies for co-immunoprecipitation included anti-TRAF2, anti-Flag and anti-pRb.

Co-immunoprecipitation efficiency was calculated by normalizing the band intensities of co-immunoprecipitated proteins to those corresponding immunoprecipitated proteins and to the expression of corresponding input lysates as previously described [50].

### Animal inoculations

Five-month-old lambs randomly allocated to three experimental groups were inoculated with either OV-IA82-Δ119 (n = 4) or OV-IA82-RV119^Flag^ (n = 4), or mock-infected (n = 3). Following anesthesia, the mucocutaneous junction of the right lower lip near the labial commissure was scarified along a two-centimeter line, and virus inoculum (0.5 ml) containing 10^7.5^ TCID50/ml was applied topically using cotton swabs. In addition, the inner sides of hind limbs were scarified in a five-centimeter line and inoculated as above. Animals were monitored for 21 days for the presence of characteristic orf lesions. Pictures were taken of the labial inoculation sites at days 3, 5, 9, 12, 16 and 21 p.i. and the lesion sizes measured with a ruler. Skin biopsy specimens from hind limb inoculation sites were collected at days 2, 5, 8, 12 and 21 p.i. fixed in 10% buffered formalin, embedded in paraffin, sectioned, and stained with hematoxylin and eosin using standard methods.

### Ethics statement

All animal procedures were approved by University of Nebraska-Lincoln Institutional Animal Care and Use Committee (IACUC; protocol 1318) and were performed in accordance with the Guide for the Care and Use of Agricultural Animals in Agricultural Research and Teaching.

### Accession numbers of virus strains used in ORFV119 sequence analysis

PPV ORFV119 amino acid sequences were aligned using Clustal Omega (EMBL-EBI). ORFV GeneBank accession numbers are (virus strains in parentheses) AY386263 (OV-IA82), AAP89015 (Orf11), ABA00637 (NZ2), 9AHH34303 (B029), ADY76823 (D1701), NP957896 (OV-SA00), AKU76741 (OV-GO), AKU76609 (OV-YX), AHZ33817 (NA1/11) and KP010356 (OV-SJ1). PCPV and BPSV GeneBank accession numbers are AEL20654 (PCPV F00-120R), AEO18268 (PCPV It1303/05), NP958027 (BPSV BV-AR02) and KM875471 (BPSV BV-TX09c5).

## Results

### ORFV119 is a nonessential early LxCxE motif-containing protein

**Fig 1:**
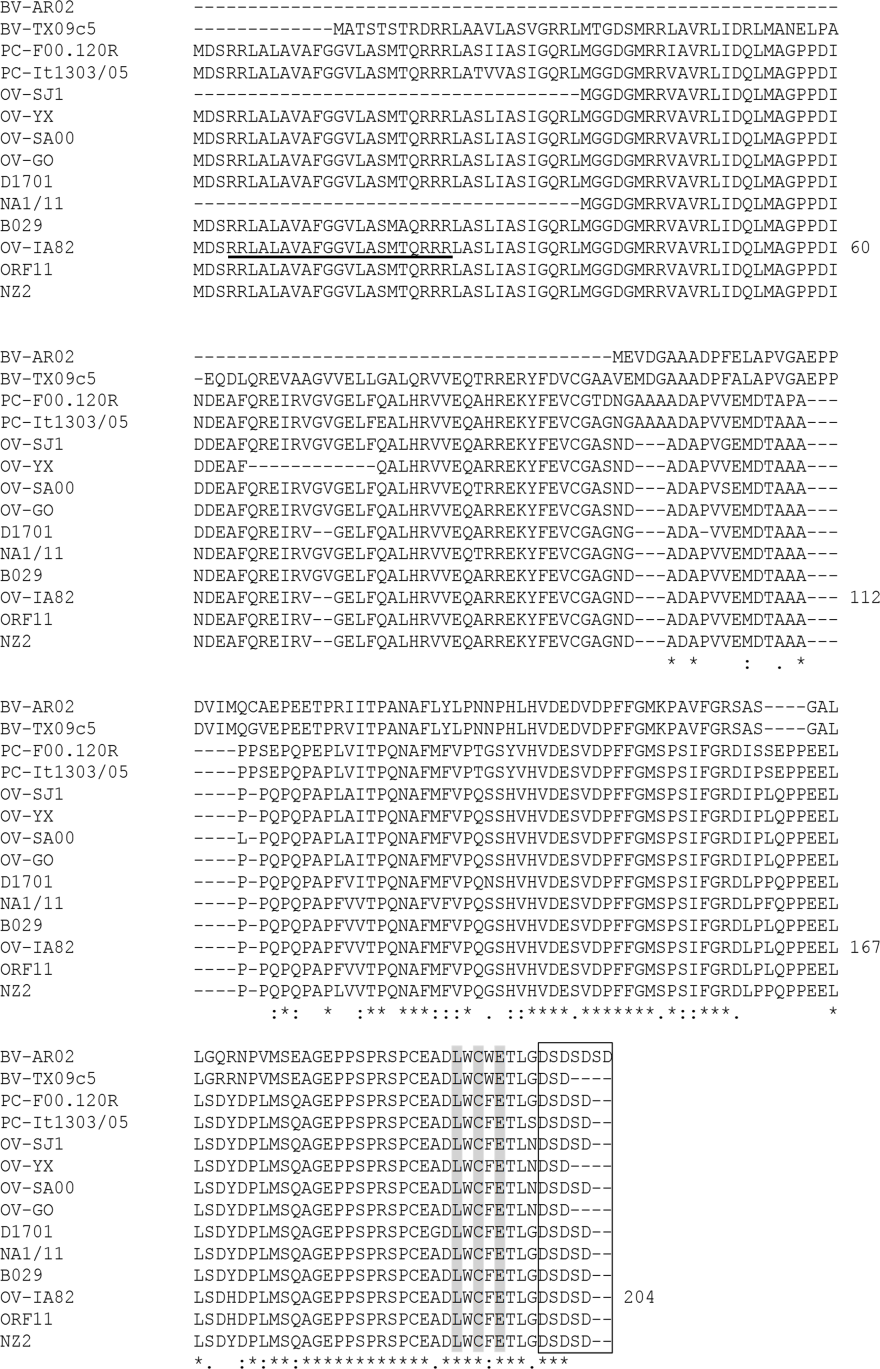
Clustal W alignment of PPV119 amino acid sequences. Aligned sequences are ORFV strains OV-IA82, NZ2, OV-SJ1, OV-XY, OV-SA00, OV-GO, D1701, NA1/11, ORF11, and B029, PCPV strains F00.120R and It1303/05, and BPSV strains BV-AR02 and BV-TX09c5 (see Materials and Methods for accession numbers). Numbers on the right indicate amino acid positions for OV-IA82 strain ORFV119. Shaded columns, boxed sequences and the underlined sequence correspond to the conserved LxCxE motif, the acidic-rich region, and the predicted mitochondrial localization signal, respectively. Asterisks [*], colons [:], and periods [.] below the alignment indicate fully, strongly, and weakly conserved, residues, respectively.

ORFV119 encodes for proteins of 170 to 206 amino acids, with predicted molecular weights of 18.6 to 22.2 kDa and percentage amino acid identities ranging from 77% to 100% (Fig 1). Homologs in Pseudocowpox virus (PCPV) and Bovine papular stomatitis virus (BPSV) are 89% and 54–56% identical to OV-IA82 ORFV119, respectively, whereas no ORFV119 homologue was found in the HL953 strain of parapoxvirus of red deer in New Zealand [51]. ORFV119 lacks homology to known proteins outside the PPV genus, and domains suggestive of protein function. However, a LxCxE motif (OV-IA82 ORFV119 positions 192–196) and a downstream stretch of acidic amino acids usually found in LxCxE motif-containing proteins are located at the C-terminus of the protein and highly conserved in PPV119 proteins (Fig 1). The LxCxE motif is required by several cellular and viral proteins to interact with members of the retinoblastoma family of proteins, which controls important aspects of cell physiology, including cell cycle progression, differentiation, and apoptosis [52]. The ORFV119 LxCxE motif and surrounding sequences follow the pattern XLXCXEXXX, where X should not be a positively charged amino acid (e.g. Lys or Arg) and X should preferably be a hydrophobic residue, which is predicted to bind pRb with high affinity [46]. A predicted mitochondrial localization sequence is found in the N-terminus of most ORFV strains and in PCPV (underlined in Fig 1), but not in BPSV119.

**Fig 2:**
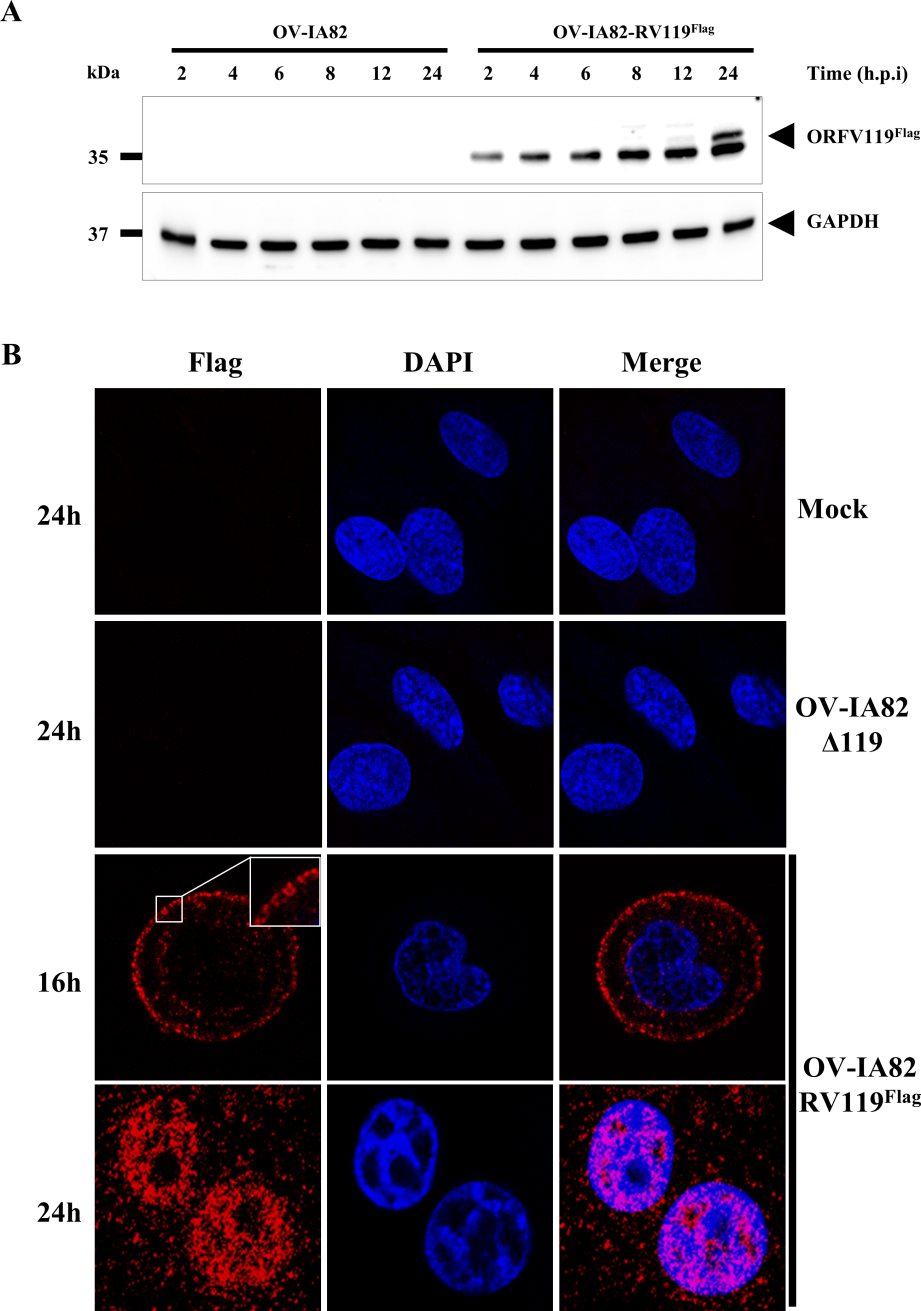
(A) Kinetics of ORFV119 expression. OFTu cells infected with OV-IA82 or revertant virus OV-IA82-RV119^Flag^ (MOI, 10) were harvested at indicated times p.i. Total cell protein extracts were resolved by SDS-PAGE, blotted and incubated with antibodies against Flag and GAPDH. Results are representative of two independent experiments. (B) ORFV119 subcellular localization. OFTu cells were mock infected or infected with OV-IA82-Δ119 or OV-IA82-RV119^Flag^ (MOI, 10), fixed at indicated times p.i. sequentially incubated with mouse monoclonal primary antibody against Flag and Alexa flour 594 labeled secondary antibody, counterstained with DAPI and examined by confocal microscopy. Insets show magnified areas of the field. ORFV119 staining is highlighted within the white box at 16 h p.i.

The kinetics of ORFV119 was assessed during ORFV replication in OFTu cells by Western blot using a recombinant virus expressing N-terminally Flag-tagged ORFV119 (OV-IA82-RV119^Flag^). A protein of approximately 32 kDa was detected at 2 h p.i. with increasing protein levels observed at later time points. At 24 h p.i., the last time point investigated, an additional slightly higher molecular weight species of ORFV119 (approximately 35 kDa) was detected (Fig 2A). The observed protein molecular weight was approximately 10 kDa higher than predicted, suggesting that the protein is post-translationally modified in some manner. Previous reports on ORFV replication have shown that early genes were expressed as early as 1 h p.i., with late genes expressed between 6 to 12 h p.i [35–38]. Newly replicated viral DNA was first detectable at 4 to 6 h p.i. accumulating rapidly between 8 and 16 h p.i [53]. Infectious virus was detected between 16 and 18 h p.i., with continuous virus production until 40 h p.i. [53]. Thus, ORFV119 is a early viral protein.

**Fig 3:**
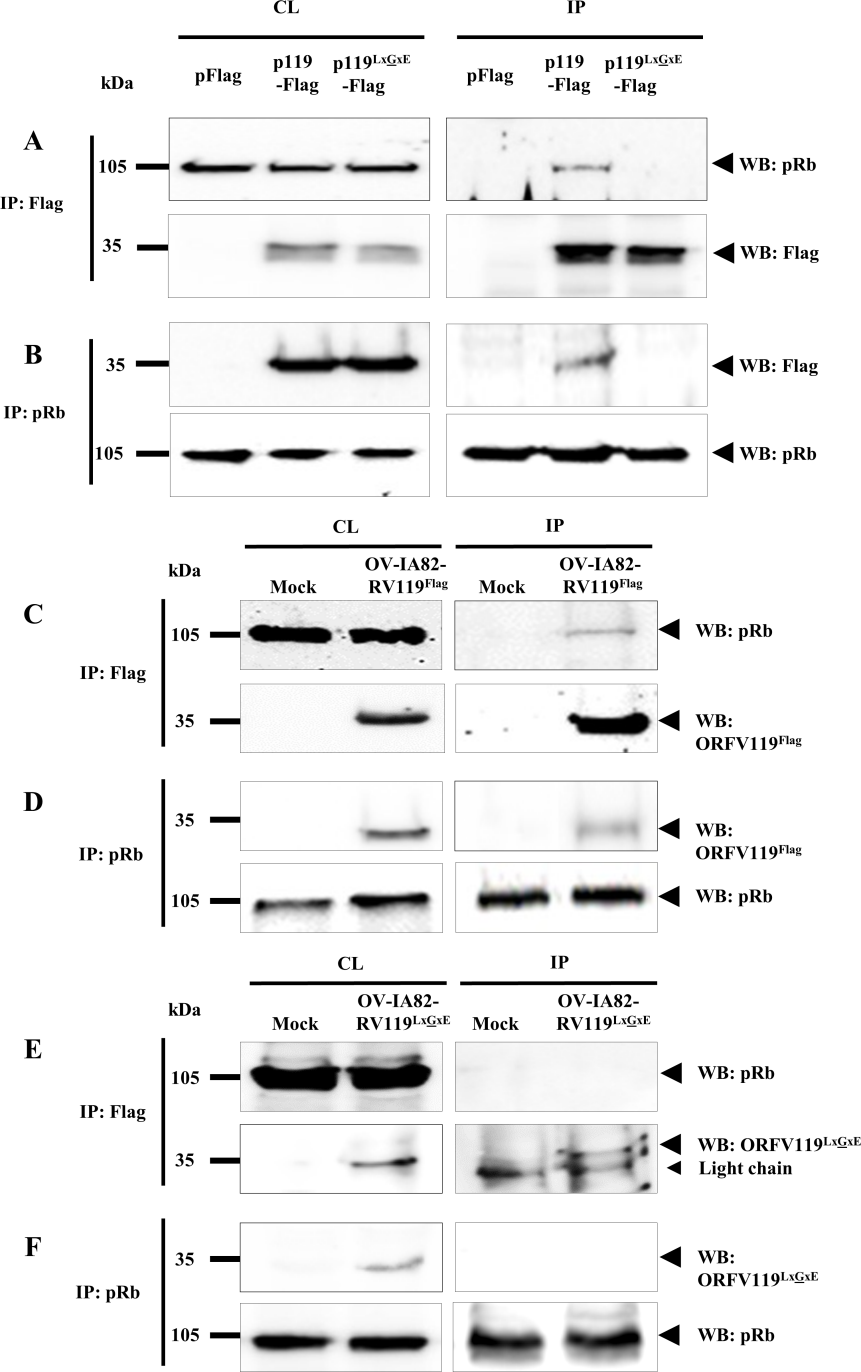
ORFV119 interacts with pRb. (A and B). 293T cells were transfected with plasmids pORFV119^Flag^ (p119^Flag^), pORFV119^LxGxE-Flag^ (p119^LxGxE-Flag^) or control plasmid (pFlag) and harvested at 12 h post transfection. Total cell lysate (CL) and protein extracts immunoprecipitated (IP) with anti-Flag (A) or anti-pRb (B) antibodies were examined by Western blot (WB) with antibodies against Flag or pRb. Results are representative of four independent experiments. (C-F) OFTu cells were mock infected or infected with OV-IA82-RV119^Flag^ (C and D), OV-IA82-RV119^LxGxE-Flag^ (E and F) (MOI, 10) and harvested at 12 h p.i. Total cell lysate and protein extracts immunoprecipitated with anti-Flag (C and E) or anti-pRb (D and F) antibodies were examined by Western blot with antibodies against Flag or pRb. Results are representative of three independent experiments. Percentage of (A) pRb co-immunoprecipitated by ORFV119^Flag^: 16.7±1.2%; (B) ORFV119^Flag^ co-immunoprecipitaed by pRb: 28.4±2.3%; (C) pRb co-immunoprecipitated by ORFV119^Flag^ in OV-IA82-RV119^Flag^ infected cells: 8.7±1.4%; (D) ORFV119^Flag^ co-immunoprecipitated by pRb in OV-IA82-RV119^Flag^ infected cells: 18.5±1.91%.

OV-IA82-Δ119, a virus lacking the *ORFV119* gene, exhibited growth kinetics and virus yields comparable to those of wild type OV-IA82 and revertant OV-IA82-RV119^Flag^ viruses in single step growth curves in OFTu cells, indicating that the gene is non-essential for growth in these cells (S1 Fig).

To examine the intracellular localization of ORFV119, OFTu cells were infected with OV-IA82-RV119^Flag^ and examined by confocal microscopy at various times post-infection. ORFV119 staining was not evident at 3 and 6 h time points. At 12 h p.i., weak punctate ORFV119 staining was observed in the cytoplasm and adjacent to the plasma membrane. By 16 h p.i., enhanced ORFV119 staining was evident, and fluorescent circular to ovoid structures (389±30nm) were observed in the cytoplasm and especially in close proximity to the cell membrane. Remarkably, at late times p.i. (24 h), OV-IA82-RV119^Flag^ infected cells exhibited abundant ORFV119 nuclear staining (Fig 2B). Fluorescence was specific for Flag-tagged ORFV119 as no signal was observed in cells infected with OV-IA82-Δ119 at all examined times p.i. (Fig 2B).

### ORFV119 interacts with the retinoblastoma protein (pRb) in an LxCxE motif-dependent manner

To investigate whether ORFV119 interacts with retinoblastoma protein pRb, 293T and HeLa cells were transfected with plasmids pORFV119^Flag^ (ORFV119^Flag^), pORFV119^LxGxE-Flag^ (ORFV119^LxGxE^) or control plasmid (pFlag), and protein extracts were prepared at 12 h post-transfection. Reciprocal co-immunoprecipitation assays with either anti-Flag or anti-pRb antibodies demonstrated that ORFV119 co-immunoprecipitates with pRb in both cell types. Co-immunoprecipitation of ORFV119 and pRb, however, was not observed with pORFV119^LxGxE-Flag^, a plasmid encoding ORFV119 in which C in the LxCxE motif was replaced by G, a change shown to abrogate interaction with pRb (Figs 3A and 3B, S2) [54]. To confirm the interaction in the context of the virus infection, OFTu cells were mock-infected or infected with OV-IA82-RV119^Flag^ or OV-IA82-RV119^LxGxE-Flag^ (MOI, 10), and cell lysates prepared at 12 h p.i. Reciprocal co-immunoprecipitation with either anti-Flag or anti pRb antibodies showed that ORFV119 but not ORFV119^LxGxE-Flag^ co-immunoprecipitates with pRb (Fig 3C–3F). Together, these results indicate that ORFV119 directly or indirectly interacts with pRb. The observation that the integrity of the LxCxE motif is required for the interaction further suggests that ORFV119 might directly bind pRb.

### ORFV119 inhibits NF-κB-signaling and NF-κB-p65 nuclear translocation early during infection

**Fig 4:**
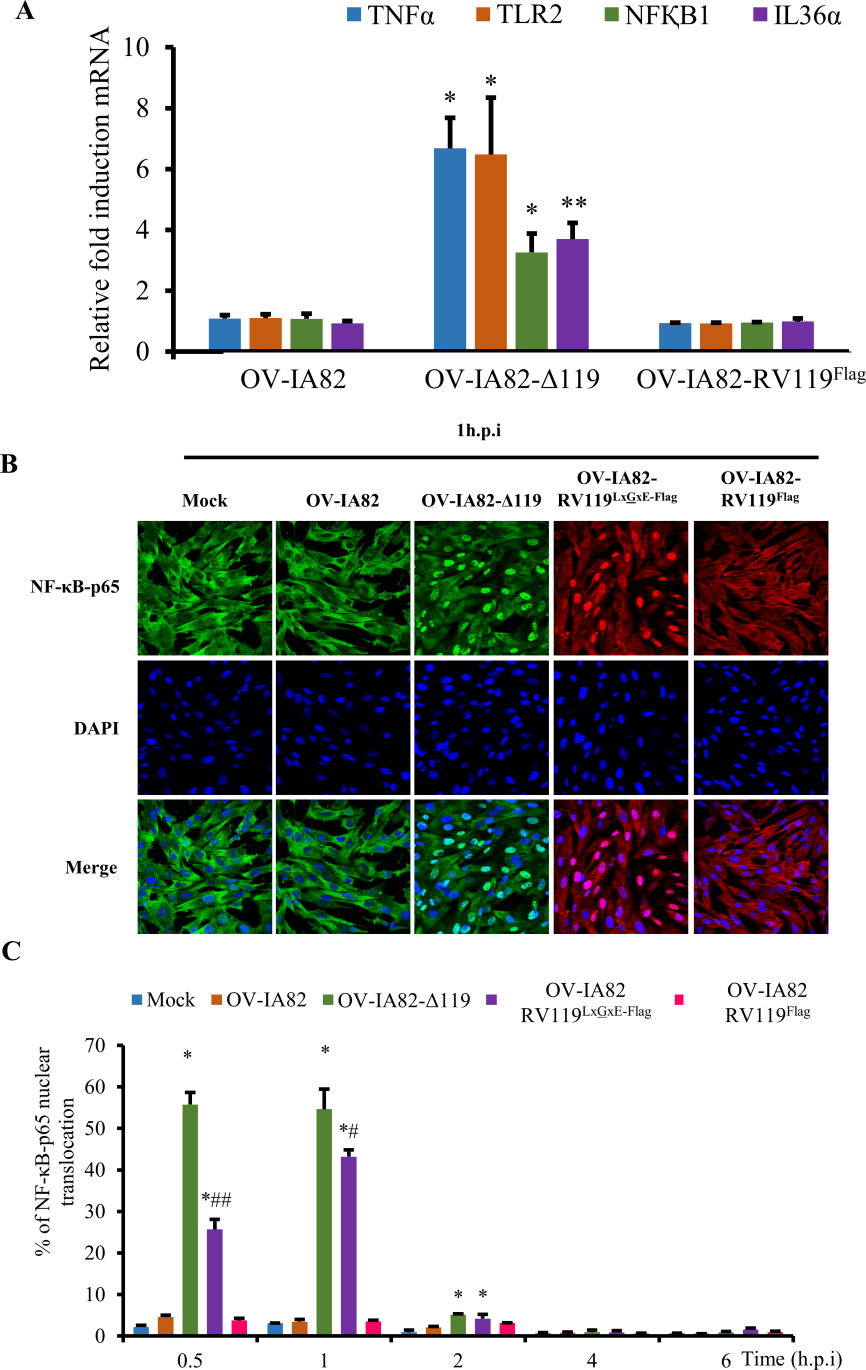
(A) Effect of ORFV119 on NF-κB mediated gene expression. OFTu cells were infected with OV-IA82, OV-IA82-Δ119, or OV-IA82-RV119^Flag^ (MOI, 10), and levels of TNFα, TLR2, NF-κB1 and IL36α mRNA assessed by real-time PCR at 2 h p.i. Fold changes are relative to OV-IA82 treatment. Results are the mean values of three independent experiments. (*, *P<0*.*05*; **, *P<0*.*01*). (B)—Effect of ORFV119 on NF-κB-p65 phosphorylation and nuclear translocation. OFTu cells were mock infected or infected with OV-IA82, OV-IA82-Δ119, OV-IA82-RV119^Flag^, or OV-IA82-RV119^LxGxE-Flag^ (MOI, 10), fixed at 1 h p.i, sequentially incubated with primary antibody against NF-κB-p65 and appropriate Alexa Fluor 488 or 594 labeled secondary antibodies, counterstained with DAPI, and examined by confocal microscopy. Green/Red, NF-κB-p65; Blue, DAPI. Results are representative of three independent experiments. (C) OFTu cells were infected and processed as in B, and the percentage of cells exhibiting nuclear NF-κB-p65 calculated at the indicated time points p.i. Results are shown as mean ± S.D. (n = 300 cells/slide) for three independent experiments [*, *P<0*.*05* (OV-IA82 vs OV-IA82-Δ119 and OV-IA82 vs OV-IA82-RV119^LxGxE-Flag^), ^#^*P<0*.*05*, ^##^*P<0*.*01* (OV-IA82-Δ119 Vs OV-IA82-RV119^LxGxE-Flag^)].

Preliminary RNA-Seq experiments indicated increased transcription of multiple NF-κB regulated genes in cells infected with OV-IA82-Δ119 compared to cells infected with OV-IA82 virus, suggesting that ORFV119 inhibits NF-κB signaling. To rule out any confounding effect from cytokines that potentially might be present in the virus inocula, viruses used in these studies were semi-purified as described in Materials and Methods. Real-time PCR analysis of gene expression showed increased levels of NF-κB-regulated genes TNFα (6.68-fold), TLR2 (6.48-fold), NF-κB1 (3.26-fold) and IL36α (3.7-fold) in cells infected with OV-IA82-Δ119 compared to OV-IA82 at 2 h p.i (Fig 4A).

To assess the effect of ORFV119 on NF-κB-p65 nuclear translocation, OFTu cells were mock infected or infected with OV-IA82, OV-IA82-Δ119, OV-IA82-RV119^LxGxE-Flag^ or OV-IA82-RV119^Flag^ and NF-κB-p65 localization was examined by immunofluorescence. Infection with OV-IA82-Δ119 and OV-IA82-RV119^LxGxE-Flag^ but not OV-IA82 or OV-IA82-RV119^Flag^ led to rapid nuclear translocation of NF-κB-p65 as early as 30 min p.i. (Fig 4B and 4C). The effect was transient as the percentage of cells expressing nuclear NF-κB-p65 returned to those in wild type and revertant virus-infected cells between 1 and 2 h p.i. (Fig 4C, *P<0*.*05*). Notably, levels of NF-κB-p65 nuclear translocation observed for OV-IA82-RV119^LxGxE-Flag^ were significantly reduced compared to those observed with OV-IA82-Δ119 (Fig 4C). Consistent with the nuclear translocation kinetics, levels of phosphorylated NF-κB-p65 (Ser536), which accumulates in the cytoplasm prior to nuclear translocation, were increased at early times p.i. (30 min and 1 h) with OV-IA82-Δ119 (S3 Fig). Together, these data show that ORFV119 is a poxviral NF-κB inhibitor acting transiently very early in infection and that ORFV119 LxCxE motif is important for the full inhibitory activity of the protein.

To explore the possibility that pRb transcriptional activity is involved in ORFV119 inhibition of NF-κB signaling in virus infected cells, we examined E2F-mediated gene transcription early in infection. OFTu cells were transfected with a plasmid encoding for a firefly luciferase reporter gene under the control of a E2F-responsive promoter and at 24 h post transfection cells were mock infected or infected with OV-IA82, OV-IA82Δ119 or OV-IA82-RV119^LxGxE-Flag^. Luciferase activities were measured at 1, 2, 4 and 6 h p.i. Similar low luciferase activity was observed in mock and virus-infected cells at 1, 2 and 4 h p.i. Significantly higher luciferase activity was observed in virus infected cells at 6 h p.i.; however, no significant difference was observed among the three viruses (S4 Fig). Thus, data suggest that ORFV119 mediated NF-κB inhibition does not involve E2F-mediated gene transcription early in infection.

### Infection with OV-IA82Δ119 results in increased phosphorylation of IKKα/β, IκBα and NF-κB-p65 early in infection

**Fig 5:**
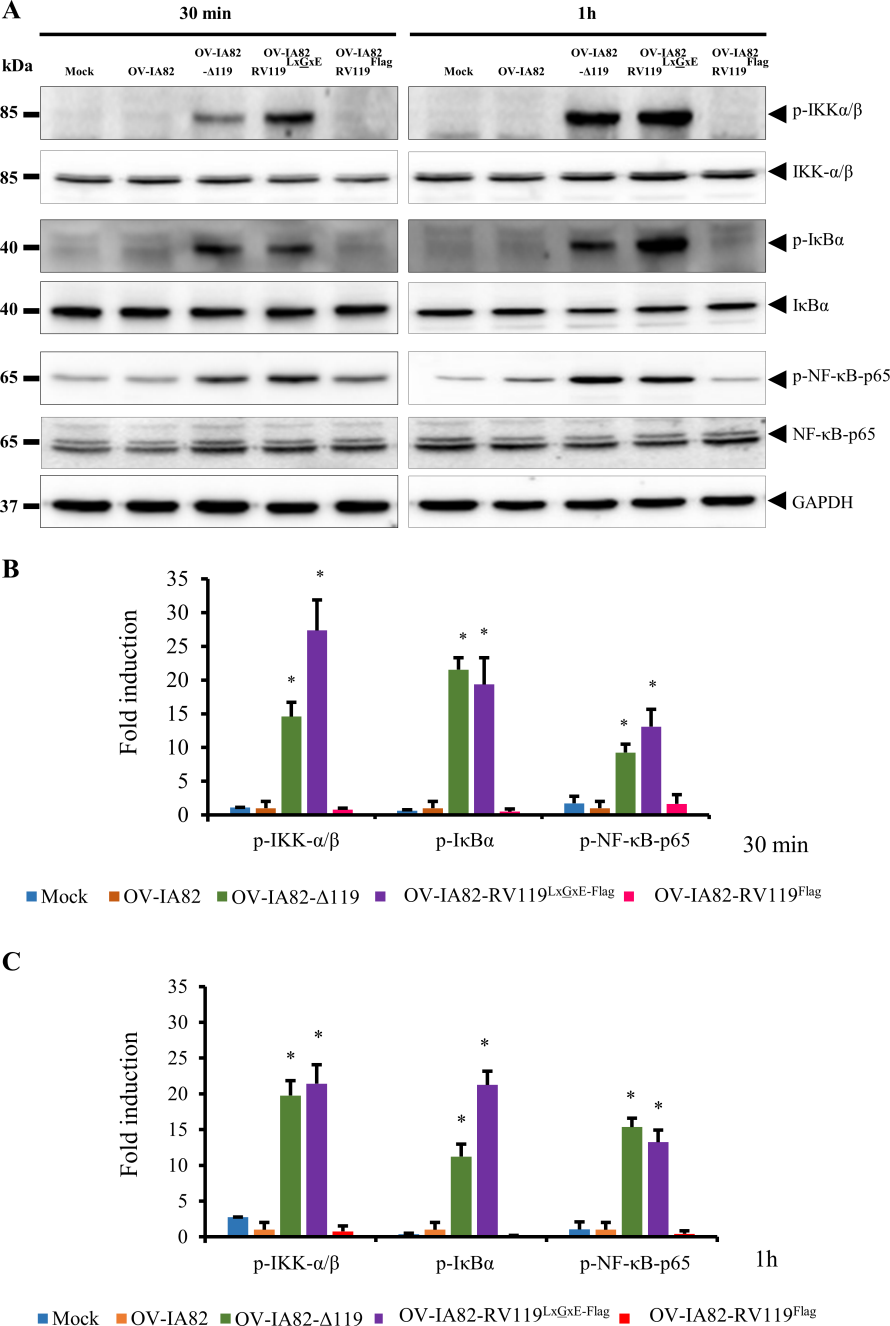
Effect of ORFV119 on IKKα/β, IκBα and NF-κB-p65 phosphorylation. (A) OFTu cells were mock infected or infected with OV-IA82, OV-IA82-Δ119, OV-IA82-RV119^LxGxE-Flag^ or OV-IA82-RV119^Flag^ viruses (MOI, 10) and harvested at the indicated times p.i. Total cell protein extracts (50 μg) were resolved by SDS-PAGE, blotted, and probed with specific antibodies shown on the right. Results are representative of three independent experiments. (B and C) Densitometric analysis of bands corresponding to phosphorylated IKKα/β, IκBα and NF-κB-p65 at 30 min (B) and 1 h p.i. (C). Densitometry of p-IKKα/β, p-IκBα and p-NF-κB-p65 bands were normalized to the loading control GAPDH. Fold changes are shown relative to OV-IA82 treatment and results are mean values of three independent experiments. (*P < 0.*05*) (OV-IA82 vs OV-IA82-Δ119 and OV-IA82 vs OV-IA82-RV119^LxGxE-Flag^).

To further investigate the mechanism of ORFV119 in NF-κB inhibition, OFTu cells were mock-infected or infected with OV-IA82, OV-IA82-Δ119, OV-IA82-RV119^LxGxE-Flag^ or OV-IA82-RV119^Flag^ for 30 min or 1 h p.i. and phosphorylation of IKKα/β, IκBα and NF-κB-p65 was assessed by Western blot. Infection by OV-IA82Δ119 and OV-IA82-RV119^LxGxE-Flag^ led to marked and early phosphorylation of IKKα/β (Ser176/180), IκBα (Ser32/36) and NF-κB-p65 (Ser536) compared to OV-IA82 infected cells (Fig 5A). In OV-IA82Δ119-infected cells, relative fold increases of phosphorylated IKKα/β (14.5 and 19.7 fold), IκBα (21.5 and 11.23 fold) and NF-κB-p65 (9.24 and 15.35 fold) were observed at 30 min and 1 h p.i., respectively (Fig 5B and 5C). Similarly, in OV-IA82-RV119^LxGxE-Flag^-infected cells, relative fold increases of phosphorylated IKKα/β (27.35 and 21.42 fold), IκBα (19.35 and 21.24 fold) and NF-κB-p65 (13 and 13.24 fold) were observed at 30 min and 1 h p.i., respectively (Fig 5B and 5C). These results indicate that ORFV119 inhibits phosphorylation of the IKK complex, a NF-κB activating event.

### ORFV119 is a virion protein and its early NF-κB inhibitory activity does not involve *de novo* viral protein synthesis

**Fig 6:**
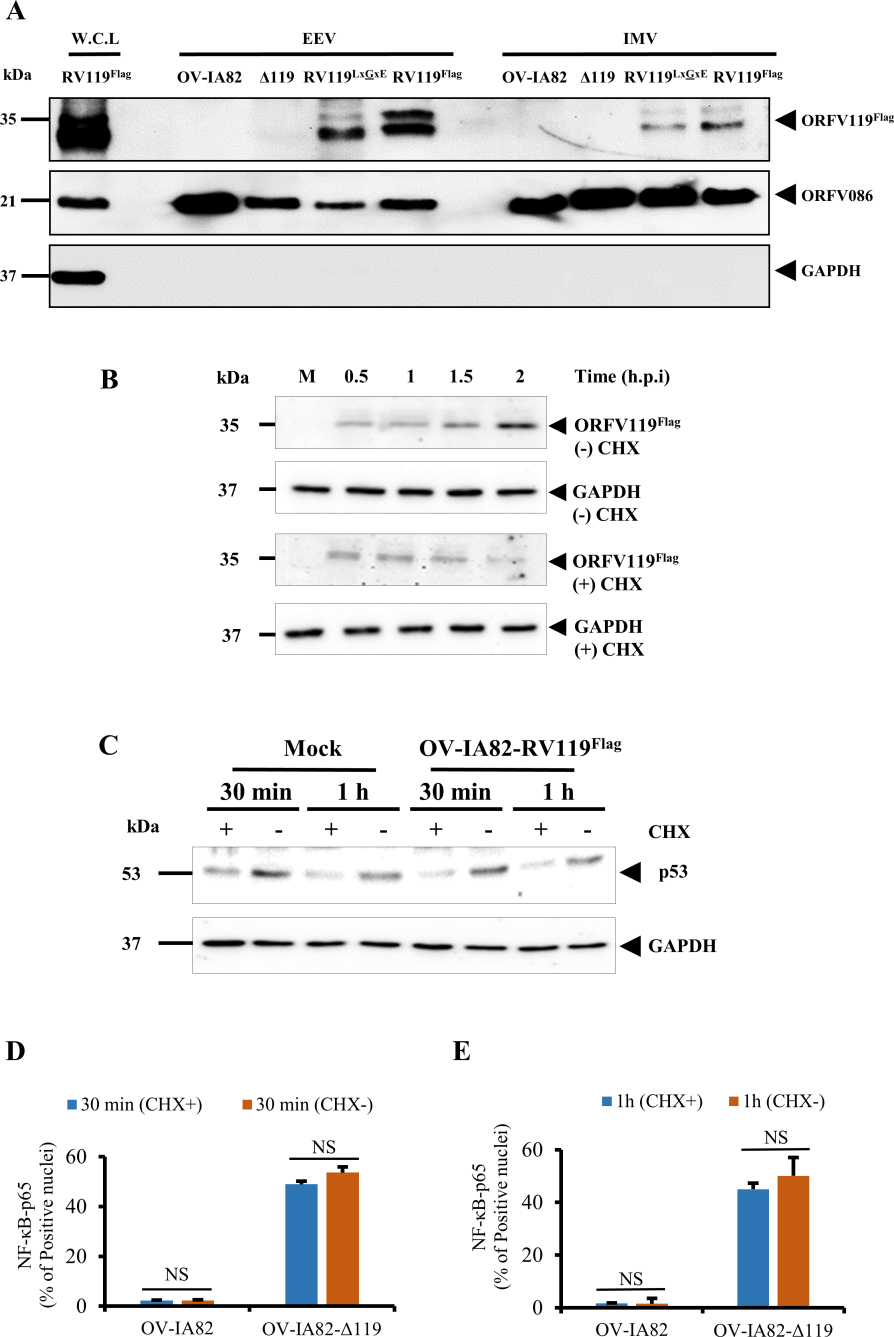
(A) Detection of ORFV119 or ORFV119^LxGxE^ in virions. OFTu cells were infected with OV-IA82, OV-IA82-Δ119 (Δ119), OV-IA82-RV119^LxGxE-Flag^ (RV119^LxGxE^) or OV-IA82-RV119^Flag^ (RV119^Flag^) and IMV and EEV virions were purified as described in Materials and Methods. Whole cell lysate (W.C.L) (60 μg) from OV-IA82-RV119^Flag^ infected OFTu cells, and purified virions (10 μg) were resolved by SDS-PAGE and analyzed by Western blot using antibodies against Flag, ORFV086 structural core protein (positive control) or GAPDH. Results are representative of three independent experiments. (B, C) Effect of translation inhibition on ORFV119 and control cellular protein p53 expression. OFTu cells mock treated or pre-treated with CHX (50 μg/ml) for 30 min were mock infected or infected with OV-IA82-RV119^Flag^ (MOI, 10) in presence of the drug and harvested at 0.5 h, 1 h, 1.5 h, and 2 h p.i. Cell extracts (50 μg) were resolved by SDS-PAGE, blotted, and probed with anti-Flag, anti-p53 and anti-GAPDH antibodies. (D, E) OFTu cells were drug treated and infected as in B with OV-IA82 or OV-IA82-Δ119 viruses, fixed at 30 min (6D) or 1 h p.i (6E), incubated with anti-NF-κB-p65 antibody, and examined by confocal microscopy. Cells were counted (n = 300 cells/slide) and results are shown as percentage of cells expressing nuclear NF-κB-p65. Results are mean values from two independent experiments. NS, non-significant by Student t test. [OV-IA82 (CHX+) vs OV-IA82 (CHX-), OV-IA82-Δ119 (CHX+) vs OV-IA82-Δ119 (CHX-)].

The observation of ORFV119 staining structures of approximate virion size in infected cells at 16 h p.i. (Fig 2B) together with the early inhibitory effect of ORFV119 on NF-κB signaling suggested ORFV119 may be a virion component available during and/or immediately after virus entry. To examine this possibility, extracellular enveloped virus (EEV) and intracellular mature virus (IMV) were purified from OFTu cells infected with an OV-IA82, OV-IA82-Δ119, OV-IA82-RV119^LxGxE-Flag^ and OV-IA82-RV119^Flag^ virus. As in infected cell extracts at 24 h p.i. (Fig 2A), western blot analysis showed a protein doublet (~32 and 35 kDa) corresponding to ORFV119^Flag^ or ORFV119^LxGxE-Flag^ in both virion fractions. As expected, the ORFV119 protein was not detected in OV-IA82 (ORFV119 lacks Flag tag) and OV-IA82-Δ119 virions (Fig 6A). A control virion core protein ORFV086 was detected as a predominant 21 kDa band in OV-IA82, OV-IA82-Δ119, OV-IA82-RV119^LxGxE-Flag^ and OV-IA82-RV119^Flag^ EEV and IMV virions [49] (Fig 6A). As a control for potential contamination of purified virions with cellular proteins, a GAPDH control was used. GAPDH protein was not detected in OV-IA82, OV-IA82-Δ119, OV-IA82-RV119^LxGxE-Flag^ and OV-IA82-RV119^Flag^ purified EEV and IMV virions (Fig 6A).

To assess whether early inhibition of NF-κB-p65 nuclear translocation by ORFV119 involves *de novo* viral protein synthesis in the infected cells, OFTu cells were pre-treated with the protein synthesis inhibitor cycloheximide (CHX) for 30 min followed by infection with OV-IA82 or OV-IA82-Δ119 for 30 min and 1 h in presence of the drug. If *de novo* synthesis of ORFV119 is required for inhibiting NF-κB signaling, then increased levels of NF-κB activation should be observed in OV-IA82 infected CHX treated cells. Low levels of ORFV119 proteins were detected at 30 min and 1 h p.i. likely representing input virion-associated protein; however, ORFV119 protein levels declined in CHX-treated OV-IA82-RV119^Flag^ infected OFTu cells at subsequent times (Fig 6B). Under these treatment conditions, expression of control host protein p53 also was inhibited (Fig 6C). Immunofluorescence analysis showed that inhibition of NF-κB-p65 nuclear translocation was unaltered in OV-IA82 infected cells regardless of CHX treatment (Fig 6D and 6E). Together, these results indicate that ORFV119 is a virion component, and suggest that virion-associated ORFV119 alone is responsible for early inhibition of NF-κB signaling.

### ORFV119 alone is sufficient for inhibiting TNFα-induced NF-κB signaling in non-infected cells

**Fig 7:**
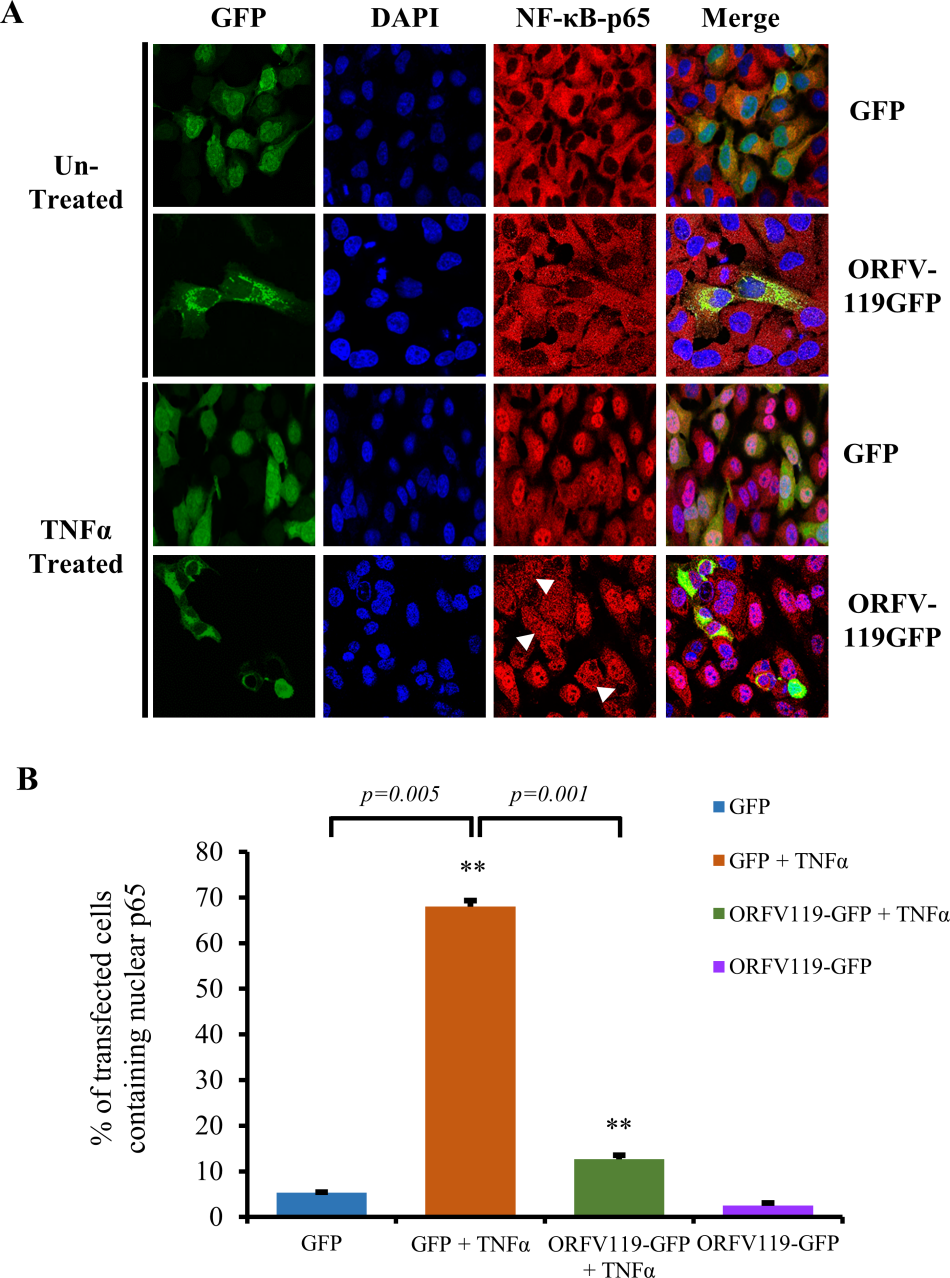
Effect of ORFV119 on TNFα-induced NF-κB-p65 nuclear translocation. (A) HeLa cells were transfected with control plasmid (pEGFP-N1) or pORFV119-GFP, which expresses ORFV119 fused to GFP. At 12 h post-transfection, cells were treated with TNFα (20ng/ml) for 30 min and processed for immunofluorescence. Green, GFP; Red, NF-κB-p65; Blue, DAPI. (B) Cells were counted (n = 500 cells/slide) and results are shown as percentage of GFP or ORFV119GFP expressing cells with nuclear NF-κB-p65. Results are mean values of three independent experiments (**P<0.*01*, GFP vs GFP + TNFα and GFP + TNFα vs ORFV119-GFP + TNFα). White arrows indicate ORFV119GFP expressing cells with reduced nuclear NF-κB-p65.

To determine if ORFV119 alone is sufficient for inhibiting TNFα-induced nuclear translocation of NF-κB-p65, immunofluorescence assays were performed in HeLa cells transiently expressing GFP or ORFV119-GFP fusion protein (119GFP). Following TNFα induction (30 min) ORFV119-GFP-expressing cells exhibited significantly reduced nuclear translocation of NF-κB-p65 (12.7%) compared to control cells expressing GFP alone (68%) (Fig 7A and 7B, *P<0*.*05*).

**Fig 8:**
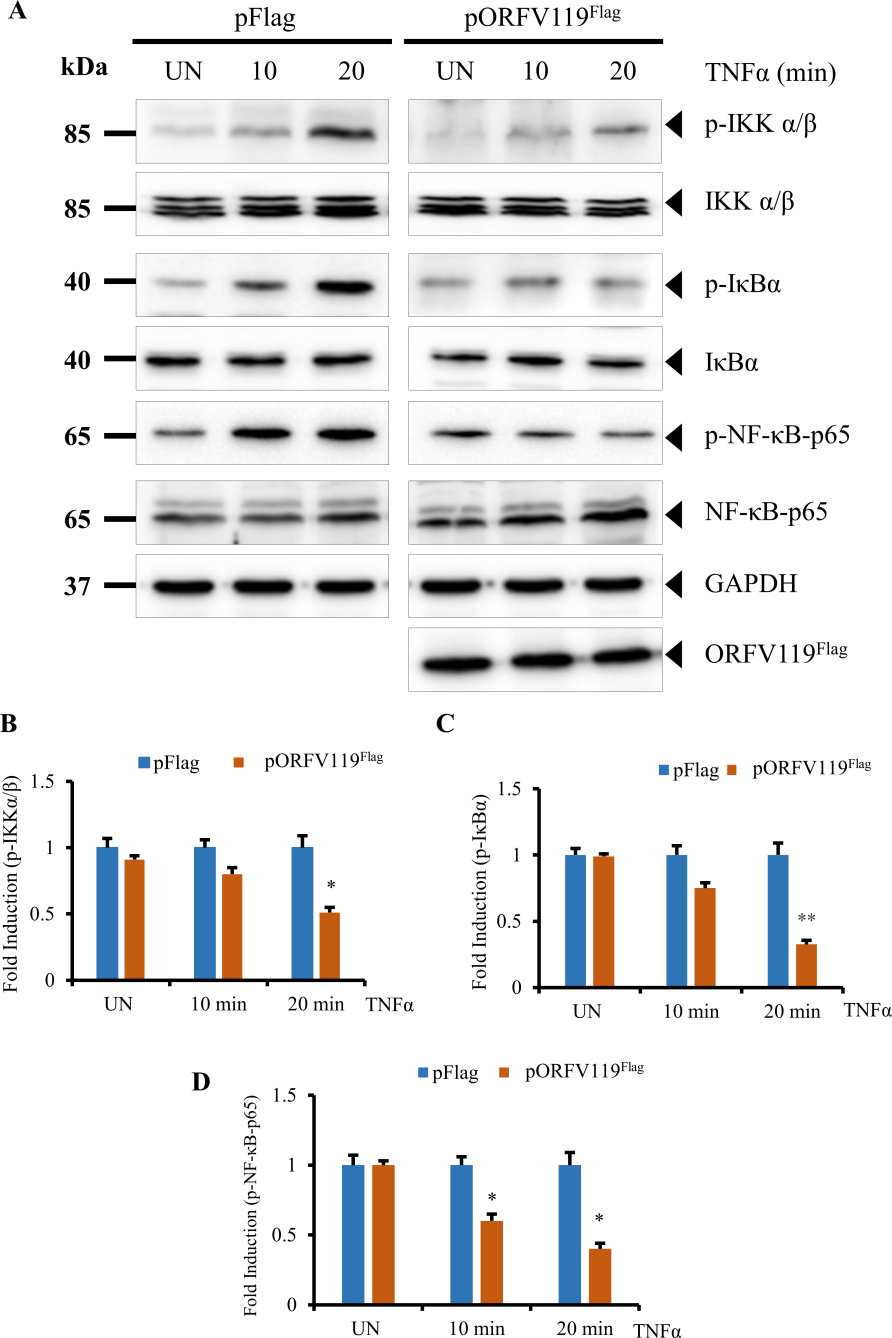
ORFV119 affects TNFα-induced NF-κB activation. (A) HeLa cells transfected with control plasmid (pFlag) or pORFV119^Flag^ were treated with TNFα (20 ng/ml) at 12 h post-transfection and harvested at the indicated time points. Total cell protein extracts (50 μg) were resolved by SDS-PAGE, blotted, and probed with specific antibodies against proteins shown on the right. (B-D) Densitometry of p-IKKα/β (B), p-IκBα (C), and p-NF-κB-p65 (D) were normalized to the loading control GAPDH. Fold changes in B-D are shown relative to the pFlag treatment. Results are mean values from two independent experiments (*P < 0.*05*, **P < 0.*01*).

ORFV119 effect on TNFα induced activation of NF-κB-p65 was further investigated by examining phosphorylation of IKKα/β (Ser176/180), IκBα (Ser32/36) and NF-κB-p65 (Ser536) in HeLa cells transfected with pFlag or pORFV119^Flag^ plasmids. ORFV119 expression markedly reduced the TNFα induced phosphorylation of IKKα/β (20 and 50%), IκBα (25 and 68%) and NF-κB-p65 (40 and 60%) in cells expressing ORFV119^Flag^ compared to control pFlag expressing cells at 10 and 20 min after TNFα induction. (Fig 8A–8D, *P<0*.*05* and *P<0*.*01*). Together, results indicate that ORFV119 inhibits TNFα-induced NF-κB signaling by preventing activation of the IKK complex in the absence of any other viral protein.

To examine if ORFV119 also affected poly(I:C), poly(A:T) or ORFV DNA-induced NF-κB transcriptional activity, HeLa cells were co-transfected with pFlag or pORFV119^Flag^ together with a plasmid encoding for a firefly luciferase reporter gene under the control of a NF-κB-responsive promoter. Cells were induced with poly(I:C), poly(A:T) or ORFV DNA at 24 h post-transfection and luciferase activities were determined at 20 h post-induction. No significant effect of ORFV119 expression on poly(I:C), poly(A:T) or ORFV DNA-induced NF-κB-mediated transcription was observed (S5 Fig). Thus, data suggest that ORFV119 functions primarily through TNFα-induced NF-κB signaling.

### pRb affects ORFV119 inhibition of NF-κB signaling

Given that: 1) ORFV119 interacts with pRb (Fig 3), 2) the ORFV119 LxCxE motif is required for that interaction (Fig 3) and 3) OV-IA82-RV119^LxGxE-Flag^—a virus containing a mutation in the ORFV119 LxCxE motif that abrogates pRb binding—was unable to efficiently inhibit NF-κB signaling (Fig 5), we examined the involvement of pRb in ORFV119-mediated inhibition of NF-κB signaling and NF-κB-p65 nuclear translocation in cells either lacking or with reduced levels of pRb.

**Fig 9:**
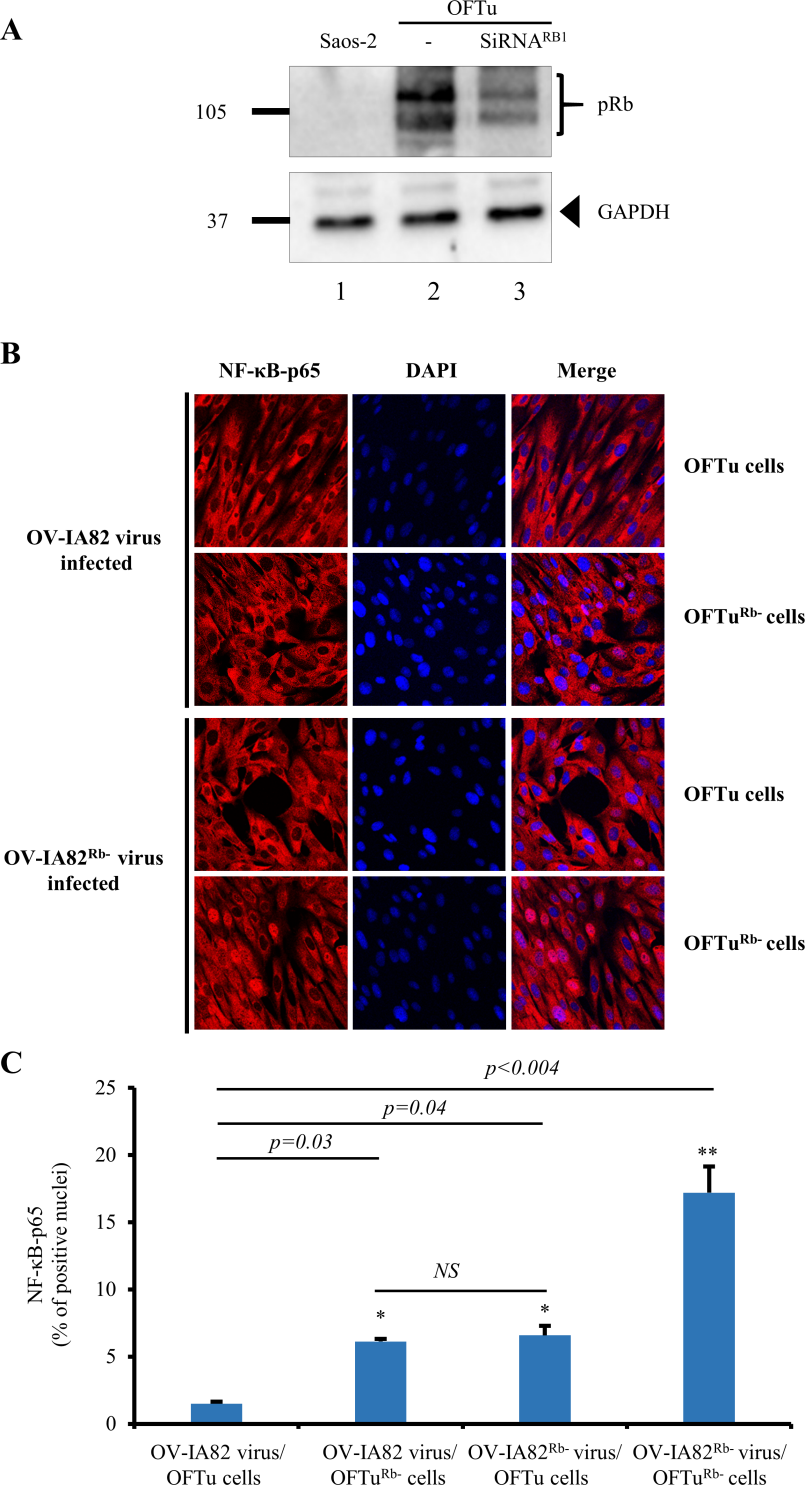
pRb affects ORFV119-mediated inhibition of NF-κB signaling in OFTu cells. (A) Western blot analysis of pRb expression in Saos-2 cells (Lane 1) and OFTu cells untreated (Lane 2) or treated with siRNA for RB1 (Lane 3) as described in Materials and Methods. Loading control, GAPDH. (B) OFTu or OFTu^Rb-^ cells were infected with OV-IA82, OV-IA82^Rb-^ virus as described in Materials and Methods and cells were fixed at 1 h p.i. sequentially probed with antibody against NF-κB-p65 and Alexa Fluor 594 labeled secondary antibody, counterstained with DAPI, and examined by confocal microscopy. Red, NF-κB-p65; Blue, DAPI. (C) Cells were counted (n = 500 cells/slide) and results are shown as percentage of cells expressing nuclear NF-κB-p65. Results are mean values from two independent experiments. *P<0.*05* (OV-IA82 virus/OFTu cells vs OV-IA82 virus/OFTu^Rb-^ cells), *P<0.*05* (OV-IA82 virus/OFTu cells vs OV-IA82^Rb-^ virus/OFTu cells, **P<0.*01* (OV-IA82 virus/OFTu cells vs OV-IA82^Rb-^/OFTu^Rb-^ cells; no significance (NS) for (C) OV-IA82 virus/OFTu^Rb-^ cells vs OV-IA82^Rb-^ virus/OFTu cells.

Cells with reduced levels of pRb (OFTu^Rb-^) were prepared from OFTu cells using siRNAs targeting ovine *RB1* (see Materials and Methods). pRb protein knockdown of approximately 60% was routinely obtained for RB1 siRNA-transfected cells at 48 h post-transfection (Fig 9A, lanes 2 and 3). Given the possibility that pRb may be associated with the virion due to its interaction with ORFV119, virus stocks were prepared in either OFTu or OFTu^Rb-^ cells (see Materials and Methods). To evaluate the effect of reduced pRb levels on ORFV119 ability to inhibit NF-κB signaling, NF-κB-p65 nuclear translocation assays were performed in OFTu or OFTu^Rb-^ cells using OV-IA82, OV-IA82^Rb-^ (OV-IA82 virus propagated in cells with reduced pRb levels), OV-IA82-RV119^LxGxE-Flag^ or OV-IA82-RV119^LxGxE-Flag-Rb-^ virus (OV-IA82-RV119^LxGxE-Flag^ virus propagated in cells with reduced pRb levels) virus stocks. As expected from data described above for OV-IA82 (Figs 4 and 5), levels of NF-κB-p65 nuclear translocation at 1 h p.i. following infection of OFTu cells using OV-IA82 virus were low (1.5% positive nuclei). However, significantly increased NF-κB-p65 nuclear translocation was observed for treatment conditions where either OFTu^Rb-^ cells (OFTu^Rb-^ cells/ OV-IA82 virus) or OV-IA82^Rb-^ virus (OFTu cells/ OV-IA82^Rb-^ virus) were used (6.1 and 6.5% NF-κB-p65 positive nuclei, respectively). Notably, using both OFTu^Rb-^ cells and OV-IA82^Rb-^ virus resulted in significantly increased levels of NF-κB-p65 nuclear translocation in infected cells (17.2% NF-κB-p65 positive nuclei) (Fig 9B and 9C). As expected for a ORFV119 protein lacking the pRb binding motif (LxCxE), no significant difference was observed in NF-κB-p65 nuclear translocation for treatment conditions where OV-IA82-RV119^LxGxE-Flag^ or OV-IA82-RV119^LxGxE-Flag-Rb-^ viruses were used to infect either OFTu or OFTu^Rb-^ cells (44.25% and 49.2% NF-κB-p65 positive nuclei, respectively) (S6 Fig). Thus, pRb contributes to the ORFV119-mediated inhibition of NF-κB signaling in infected OFTu cells.

**Fig 10:**
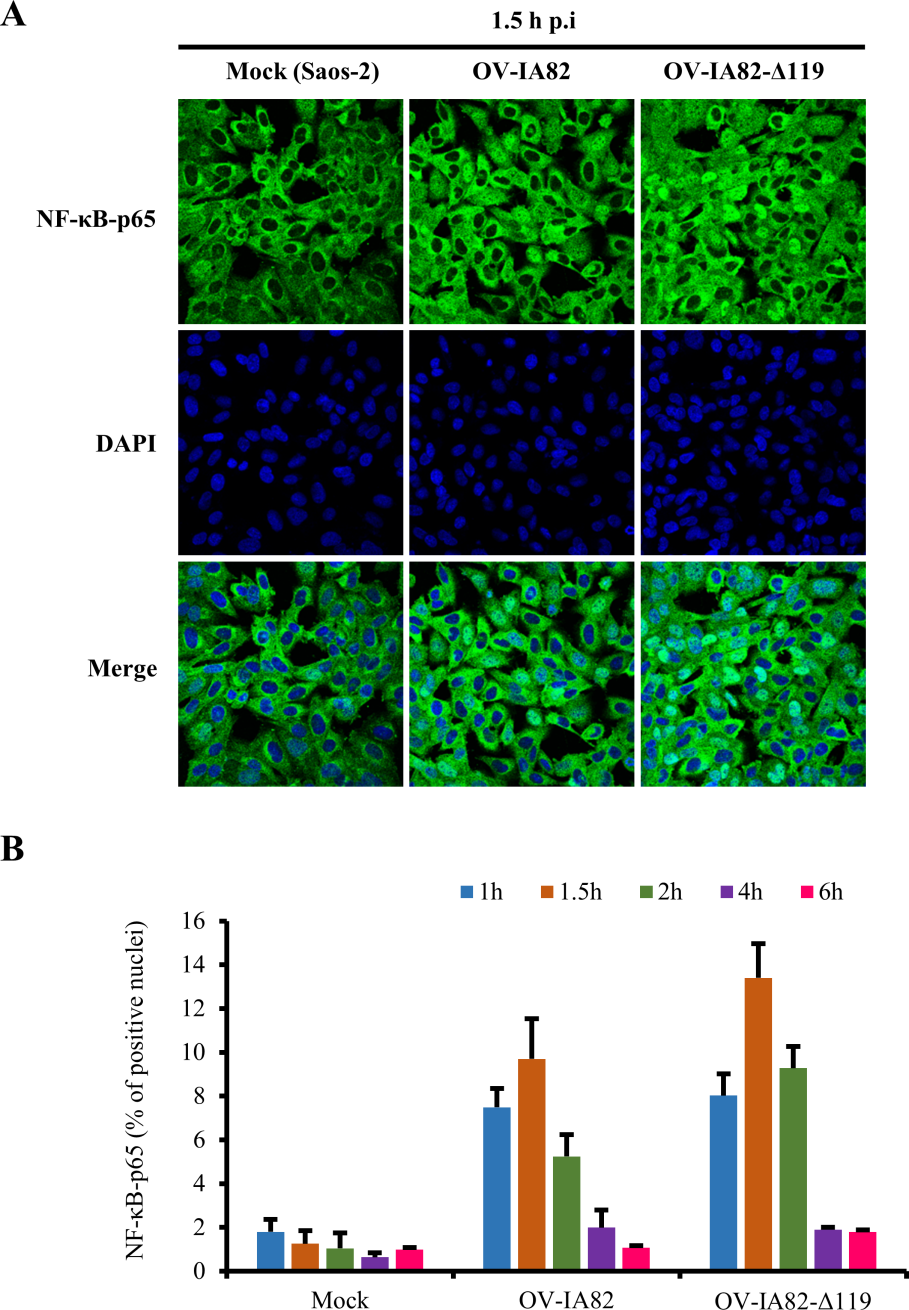
pRb affects ORFV119-mediated inhibition of NF-κB signaling in Saos-2 cells (A) Saos-2 cells were mock infected or infected with wild-type OV-IA82 or OV-IA82Δ119 (MOI, 50), fixed 1 h p.i. sequentially probed with primary antibody against NF-κB-p65 and Alexa Fluor 488 labeled secondary antibody, and counterstained with DAPI. Green, NF-κB-p65; Blue, DAPI. (B) Cells were counted (n = 500 cells/slide) and results are shown as percentage of cells expressing nuclear NF-κB-p65. Results are mean values from two independent experiments. *p* values for OV-IA82 vs OV-IA82Δ119 comparisons at all-time points were not significant.

Additional experiments examining involvement of pRb in ORFV119-mediated inhibition of NF-κB signaling were conducted in human osteosarcoma Saos-2 cells, a pRb-deficient cell line [55]. pRb was not detected in Saos-2 cell extracts by western blot. (Fig 9A, Lane1). If pRb is mediating ORFV119 inhibition of NF-κB-p65 nuclear translocation, increased NF-κB-p65 nuclear translocation would be expected in OV-IA82 virus infected Saos-2 cells. Saos-2 cells were mock infected or infected with OV-IA82 or OV-IA82-Δ119 and NF-κB-p65 localization was examined by immunofluorescence at indicated times p.i. No significant differences in NF-κB-p65 positive nuclei in OV-IA82-or OV-IA82-Δ119-infected cells were observed at any time post-infection (Fig 10A and 10B). Thus, in the absence of pRb, the early NF-κB inhibitory phenotype observed for OV-IA82 is lost. Together, data using cells with reduced pRb levels or lacking pRb altogether indicate that ORFV119-mediated inhibition of NF-κB-signaling is largely pRb-dependent.

### ORFV119 interacts with TRAF2, a component of the NF-κB signaling pathway

**Fig 11:**
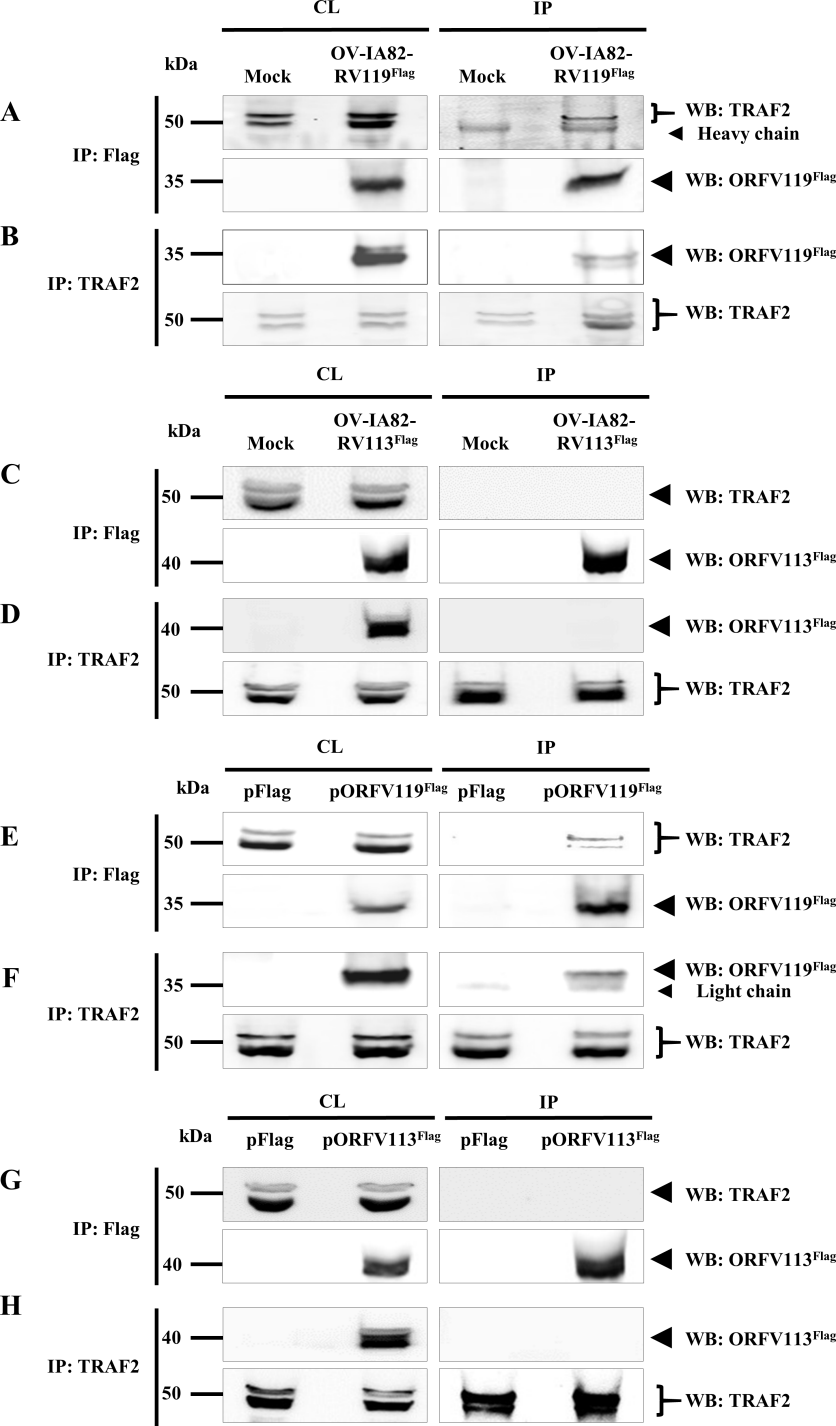
Co-immunoprecipitation of ORFV119 with TRAF2. (A-D) OFTu cells were mock infected or infected with OV-IA82-RV119^Flag^ or OV-IA82-RV113^Flag^ (MOI, 10) and harvested at 12 h p.i. Total cell lysate and protein extracts immunoprecipitated with anti-Flag (A and C) or anti-TRAF2 (B and D) antibodies were examined by Western blot using anti-TRAF2 or anti-Flag antibodies. (E-H) 293T cells were transfected with control plasmid (pFlag), pORFV119^Flag^ or pORFV113^Flag^ and harvested at 12 h post transfection. Total cell lysate and protein extracts immunoprecipitated with anti-Flag (E and G) or anti-TRAF2 (F and H) antibodies were examined by Western blot using anti-TRAF2 or anti-Flag antibodies. Results are representative of three independent experiments. Percentage of (A) TRAF2 co-immunoprecipitated by ORFV119^Flag^ in OV-IA82-RV119^Flag^ infected cells: 35.3±2.7%; (B) ORFV119^Flag^ co-immunoprecipitated by TRAF2 in OV-IA82-RV119^Flag^ infected cells: 24.7±1.25%; (E) TRAF2 co-immunoprecipitated by ORFV119^Flag^ in pORFV119^Flag^ transfected cells: 23.7±2.1%; (F) ORFV119^Flag^ co-immunoprecipitated by TRAF2 in pORFV119^Flag^ transfected cells: 18.1±2.02%.

To assess potential interactions of ORFV119 with components of the TNFα-induced NF-κB signaling pathway, co-immunoprecipitation assays were conducted with antibodies against TRAF2, TAK1, RIP1, TRAF6 and NEMO. OFTu cells were mock-infected or infected with OV-IA82-RV119^Flag^ (MOI, 10) and harvested at 12 h p.i. Total cell protein extracts were immunoprecipitated with anti-Flag or anti-target cellular protein antibodies. Reciprocal co-immunoprecipitation demonstrated that ORFV119 co-immunoprecipitates with TRAF2 (Fig 11A and 11B). Similar results were obtained in 293T cells transiently expressing pORFV119^Flag^ (Fig 11E and 11F); however, no interaction was observed between TRAF2 and transiently expressed ORFV119^LxGxE^ (S7 Fig). As a control for specificity of ORFV119 and TRAF2 interaction, a Flag tagged ORFV113^Flag^ was used. No interaction was observed between ORFV113^Flag^ and TRAF2 in OFTu cells infected with OV-IA82-RV113^Flag^ (Fig 11C and 11D) nor in 293T cells transfected with pORFV113^Flag^ (Fig 11G and 11H). In the context of viral infection, reciprocal co-immunoprecipitation of ORFV119 with TAK1, RIP1, TRAF6 and NEMO was not observed. Together, these results indicate that ORFV119 directly or indirectly interacts with the scaffold protein TRAF2. Further, dependence on a LxCxE motif for interaction suggested that pRb might play a role in ORFV119-TRAF2 complex formation.

### pRb enhances ORFV119-TRAF2 interaction

**Fig 12:**
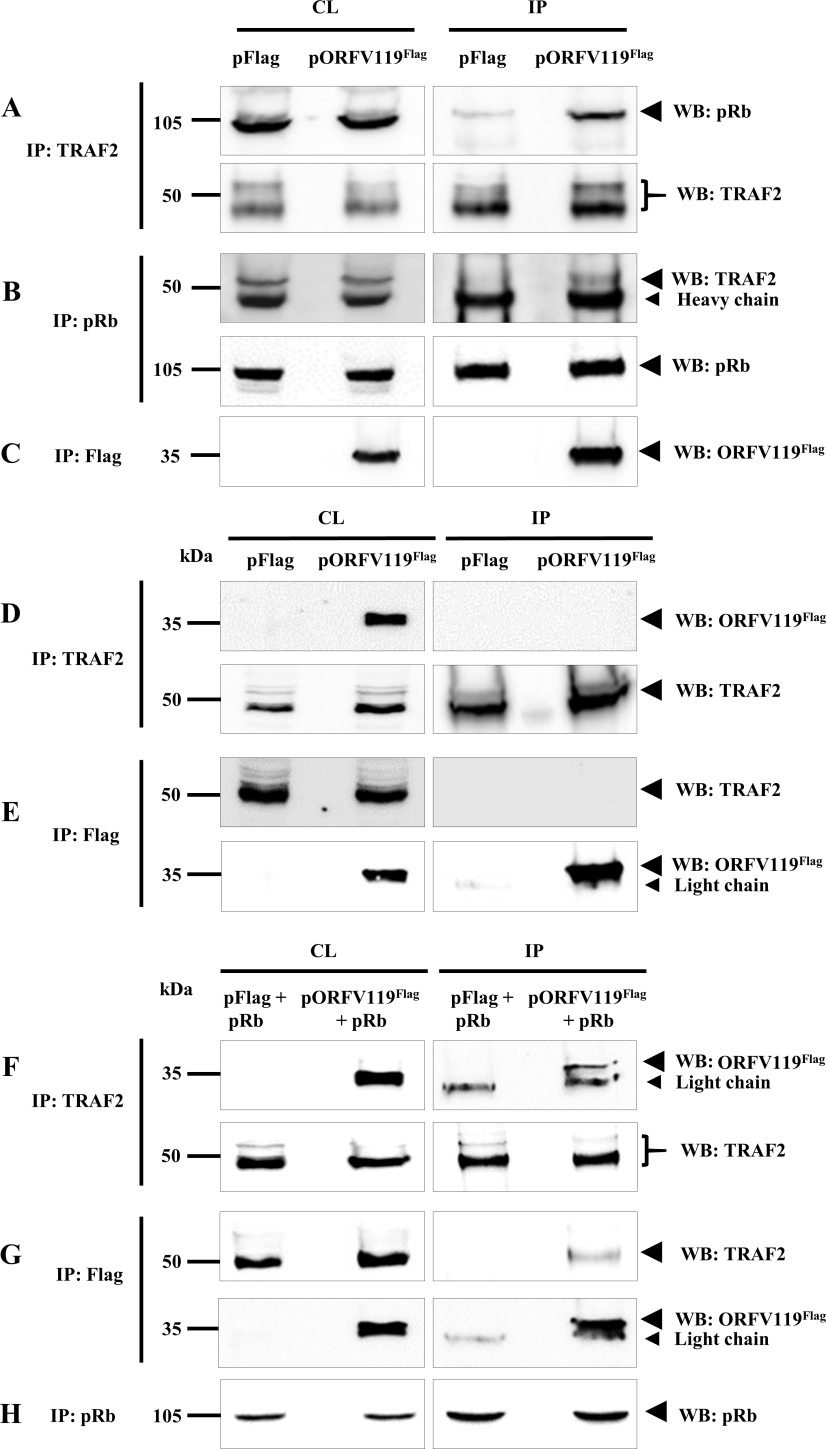
Co-immunoprecipitation of pRb with TRAF2 in 293T cells and ORFV119 with TRAF2 in Saos-2 cells. (A-C) Co-immunoprecipitation of pRb with TRAF2 in 293T cells. 293T cells were transfected with control plasmid (pFlag) or pORFV119^Flag^. Total cell lysate and proteins extracts immunoprecipitated with antibodies against TRAF2 (A), pRb (B) or Flag (C), were examined by Western blot (WB) using anti-pRb, anti-TRAF2 or anti-Flag antibodies. (D and E) Co-immunoprecipitation of ORFV119 with TRAF2 in Saos-2 cells. Saos-2 cells were transfected with control plasmid (pFlag) or pORFV119^Flag^ and harvested at 12 h post transfection. Total cell lysate and protein extracts immunoprecipitated with anti-TRAF2 (D) and anti-Flag (E) antibodies were examined by Western blot using anti-Flag and anti-TRAF2 antibodies. (F-H) Co-immunoprecipitation of ORFV119 with TRAF2 in Saos-2 cells transiently expressing ORFV119^Flag^ and pRb. Saos-2 cells were co-transfected with control plasmid (pFlag) or pORFV119^Flag^ and pRb plasmids and harvested at 12 h post transfection. Total cell lysate and protein extracts immunoprecipitated with anti-TRAF2 (F), anti-Flag (G) and anti-pRb (H) antibodies were examined by Western blot using anti-Flag, anti-TRAF2 and anti-pRb antibodies. Results are representative of two independent experiments. Percentage of (A) pRb co-immunoprecipitated by TRAF2 in pFlag transfected cells: 5.1±0.58%; pORFV119^Flag^ transfected cells: 28.4±2.73%; (B) TRAF2 co-immunoprecipitated by pRb in pFlag transfected cells: 3.1±0.53%; pORFV119^Flag^ transfected cells: 10.3±1.64%. (F) ORFV119^Flag^ co-immunoprecipitated by TRAF2 in pORFV119^Flag^ and pRb transfected cells: 15.2±1.26%; (G) TRAF2 co-immunoprecipitated by ORFV119^Flag^ in pORFV119^Flag^ and pRb transfected cells: 18.7±0.96%.

As ORFV119-mediated inhibition of the NF-κB signaling is pRb dependent (Figs 4, 5, 9 and 10) and ORFV119 interacted with TRAF2 in a LxCxE motif-dependent manner (S7 Fig), we hypothesized that a ORFV119-pRb complex may be required to efficiently interact with TRAF2. To examine this possibility, 293T cells were transfected with pFlag or pORFV119^Flag^ and harvested at 12h p.i. Reciprocal co-immunoprecipitation assays using total cellular protein extracts with either anti-TRAF2 or anti-pRb antibodies demonstrate that TRAF2 and pRb co-immunoprecipitation, while weak or absent in pFlag transfected cells, is enhanced by the presence of ORFV119 in pORFV119^Flag^ transfected cells (Fig 12A and 12B).

To further evaluate a pRb requirement for efficient ORFV119-TRAF2 interaction, co-immunoprecipitation experiments were performed in Saos-2, a pRb-deficient cell line. In contrast to results described above (Fig 11), where ORFV119-TRAF2 interaction was observed in pRb expressing OFTu and 293T cells, no interaction was detected in Saos-2 cells (Fig 12D and 12E). Notably, transfection of Saos-2 cells with both pORFV119 and pRb plasmids restored the interaction as reciprocal co-immunoprecipitation of ORFV119 and TRAF2 were observed (Fig 12F and 12G). Taken together, these data indicate that pRb is important for ORFV119-TRAF2 interaction and, further, they suggest that a ORFV119-pRb complex may be required for efficient interaction with TRAF2.

### ORFV119 affects ORFV virulence in the natural host

The contribution of ORFV119 to virus virulence was investigated in sheep, a natural ORFV host. Animals were inoculated with OV-IA82-Δ119 (n = 4), OV-IA82-RV119^Flag^ (n = 4) or PBS (control group, n = 3) in the right labial commissure and the inner side of the thighs, and disease course was monitored for 21 days. All virus-inoculated animals developed clinical orf as evidenced by erythema, papules, pustules, and scabby tissue deposition on the lips (S8A Fig). However, beginning on day 3 p.i., lesions in sheep inoculated with OV-IA82-RV119^Flag^ were 25% to 90% larger and exhibited more extensive pustules and scabby tissue deposition than those inoculated with deletion mutant virus (S8A and S8B Fig). By day 16 p.i., lesions in all sheep inoculated with OV-IA82-Δ119 were resolved. In contrast, three of four sheep inoculated with OV-IA82-RV119^Flag^ (sheep # 79, #101, and #639) still exhibited well-defined lesions at day 16 p.i. and regressing lesions still were present in two of the animals (#79 and #101) at day 21 p.i. (end point of the experiment) (S8A Fig). No lesions were observed in PBS-inoculated controls. Histological examination of punch biopsies collected from the thighs at various times post-infection showed no significant differences in time to onset and type of skin changes between groups of virus-inoculated animals. Overall, results indicate that lesion development is more restricted and time to resolution is reduced in sheep inoculated with OV-IA82-Δ119 compared to revertant virus. Thus, ORFV119 contributes to ORFV virulence in the natural host.

## Discussion

The NF-κB signaling pathway plays key roles in the skin by regulating innate immune responses, inflammation, cell survival and cell proliferation [56–58]. Crucial cytoplasmic and nuclear events in the signaling pathway are targeted by various proteins encoded by the highly epitheliotropic ORFV [35–37]. Here, we describe an ORFV protein, ORFV119, that interacts with pRb and prevents activation of NF-κB signaling very early during infection. Inhibition of NF-κB-signaling by ORFV119 is largely dependent on its ability to interact with pRb, which prevents activation of the IKK complex and downstream NF-κB signaling.

ORFV119 was shown to interact with pRb in infected and uninfected cells, indicating that other ORFV proteins are not required for the interaction. Similar to oncoproteins of small DNA viruses, binding to pRb was dependent on the ORFV119 LxCxE motif since a CxG substitution in the motif abrogated the interaction completely (Fig 3). This suggests that ORFV119, like small DNA virus oncoproteins, might directly bind pRb. Interestingly, another highly epitheliotropic poxvirus, molluscum contagiosum virus (MCV), encodes a protein (MC007L) unrelated to ORFV119 that localizes to mitochondria and interacts with pRb through an LxCxE motif [59]. The function of MC007L in MCV infection is unknown.

pRb has not been previously implicated in antiviral responses against poxviruses; however, it has been associated with NF-κB modulation by other viruses. For example, transiently expressed adenovirus E1A, a pRb-binding oncoprotein, was shown to inhibit NF-κB-dependent transcription induced by TNFα, the effect being dependent on E1A/pRb interaction [44]. And pRb was shown to be required for the activation of the NF-κB pathway in response to vesicular stomatitis virus infection, although viral mechanisms involved were not described [45].

Through association with pRb, ORFV119 inhibits NF-κB signaling by preventing IKK complex activation (Fig 5), with ORFV119-TRAF2 interaction likely underlying the inhibition (Fig 11). Results suggest that a ORFV119-pRb complex may be required for efficient interaction of ORFV119 with TRAF2, leading to inhibition of NF-κB signaling (Fig 12 and S7 Fig). TRAF2 is a RING finger protein recruited to TNF receptors to regulate NF-κB signaling both positively and negatively [60]. Viral proteins that interact with TRAF2 activate or inhibit NF-κB signaling. For example, the MCV protein MC159 was shown to interact with both TRAF2 and NEMO and to inhibit NF-κB activation [61], while Kaposi’s sarcoma associated-herpes virus (KSHV) vFLIP and rotavirus VP4 proteins interact with TRAF2 activating NF-κB signaling [62,63]. The effect of these interactions on TRAF2 function remains unknown. Conceivably, viral proteins might interfere with NF-κB signaling by modulating TRAF2 E3 ubiquitin ligase activity and/or by affecting TRAF2 scaffold functions.

**Fig 13:**
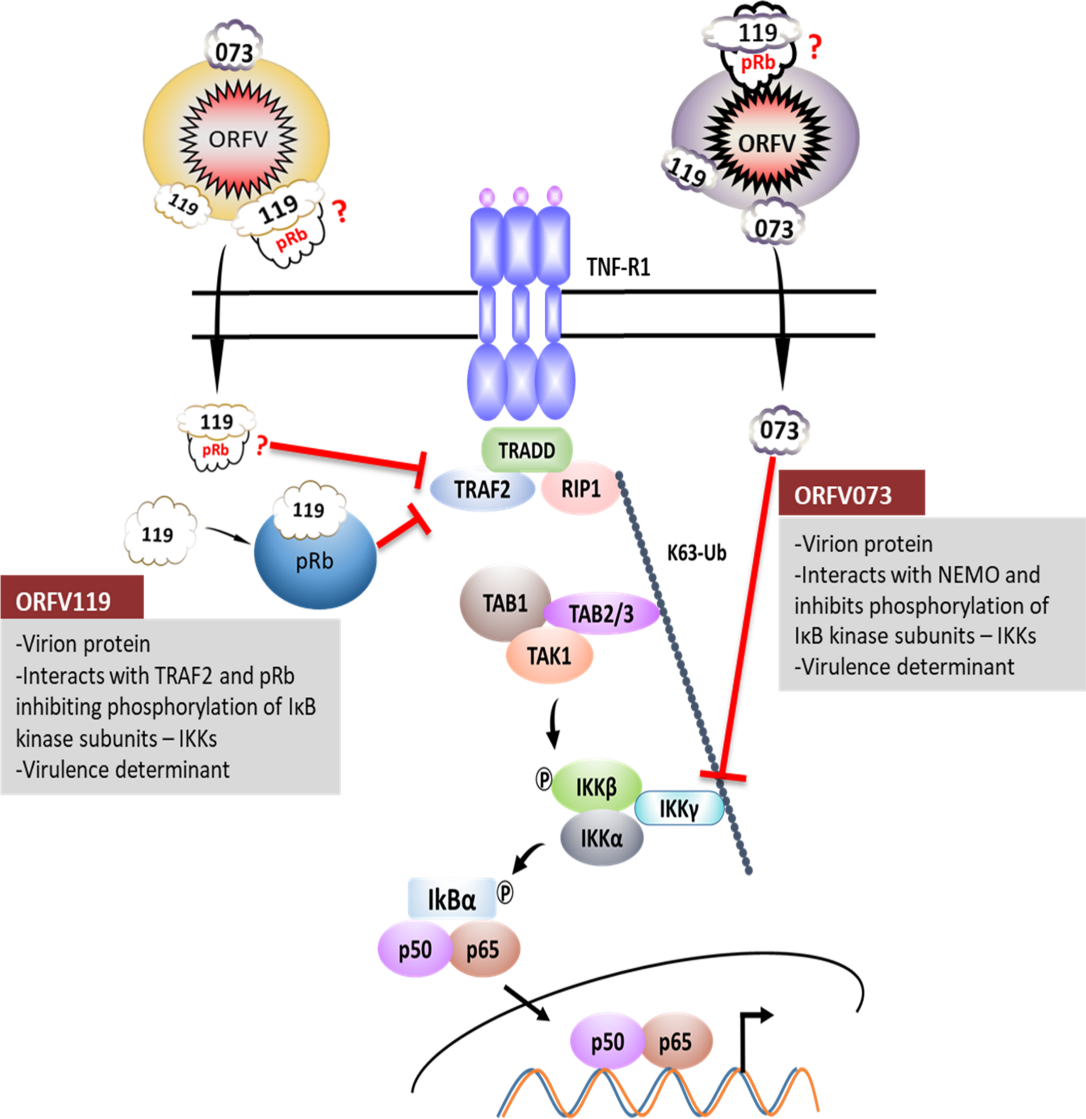
Regulation of NF-κB signaling pathway by ORFV virion associated NF-κB inhibitors. ORFV073 inhibits NF-κB signaling by preventing activation of IKK complex through interaction with NEMO, the regulatory subunit of the IKK complex. ORFV119 interacts with pRb and TRAF2 inhibiting the activity of IKK complex and downstream NF-κB signaling early in infection. Localization of pRb in virions remains to be demonstrated.

ORFV119 is a virion protein (Fig 6) functioning very early in infection (≤ 30 min) to inhibit NF-κB signaling. Observed early IKK complex activation on infection of cells with virus lacking ORFV119 (OV-IA82Δ119) indicates ORFV119 inhibits NF-κB signaling induced by an early infection event. Early inhibition of IKK complex activation during infection, ORFV119-TRAF2 interaction in infected cells, and a proposed role for TRAF2 in poxvirus entry [64] suggest that ORFV119 may be inhibiting ORFV entry mediated activation of NF-κB signaling. The importance of viral inhibition of NF-κB activation very early in ORFV infection is further supported by the presence of a second virion-associated NF-κB inhibitor ORFV073 (Fig 13). ORFV073 inhibits NF-κB signaling by preventing activation of IKK complex through interaction with NEMO, the regulatory subunit of the IKK complex [38]. As early infection events, including those related to cell entry, are likely conserved among poxviruses [65], early inhibition of NF-κB signaling in poxvirus infected cells may be of greater biological significance than currently appreciated.

Notably, at late times post-infection (≥24 h p.i) ORFV119 also is observed in the nucleus of infected cells (Fig 2), suggesting that in addition to the early virion-associated NF-κB inhibitory function described here, the protein may perform additional functions in the infected cell. The fact that most of pRb localizes to the cell nucleus raises the question as to whether ORFV119 interacts with nuclear pRb at late times post-infection affecting pRb functions such as transcriptional control and cell cycle regulation.

Other ORFV NF-κB inhibitors have been detected in the nucleus. ORFV002 localized to the nucleus and inhibited nuclear phosphorylation of NF-κB-p65 by interacting with mitogen stimulated stress kinase 1 (MSK1) [36,39]. And, ORFV073, a virion-associated NF-κB inhibitor, also was shown to localize to the nucleus yet a nuclear function for the protein has not been described [38]. Among other poxviruses, vaccinia virus protein K1 localized to the nucleus where it prevented acetylation of NF-κB-p65 [66].

Examination of published parapoxviral genomes shows that a genomic region encompassing ORFV119 is affected sporadically by gaps or deletions of as much as 5.7 kbp [67–69]. Notably, while the extent of the deletion varied considerably, ORFV119 was always affected. The genomic sequence of the PCPV F00-120R strain, for example, was found to contain a 5.5 kbp DNA deletion that removed genes 116–121 [67]. Retrospective PCR analysis, however, strongly suggested that loss of the 5.5 kbp region occurred during virus passage in tissue culture [68]. Deletions affecting this region were also found in ORFV isolates OV-NP (5.7 kbp deletion) and OV-SJ1 (1.5 kbp deletion), which were isolated from goat lesion material [69]. Although retrospective PCR analysis was not conducted, these virus isolates (cell culture-passage 6) were fully attenuated following inoculation of goats, further supporting the notion that the genomic loss occurred during early passage in cells, and indicating that important virulence functions are encoded within this genomic region. Genomic deletions resulting in complex fragmentation of the BPSV ORFV*119* homolog have also been reported [70]. An explanation for instability of this genomic region upon virus passage in cell culture is lacking; however, it is possible that loss of these genes may be advantageous for virus growth in cell culture.

Here, infection with a deletion mutant virus lacking *ORFV119* resulted in restricted lesion development and reduced time to resolution compared with revertant virus infection in the natural host. The attenuated phenotype of infection likely indicates improved host control of ORFV infection in the absence of ORFV119 (S8 Fig). Our results are at variance with a previous report where no differences in virulence and pathogenesis were observed between wild type and ORFV119 deletion viruses [71]. The discrepancy likely reflects differences in viral strains (IA82 vs SHZ1), extent of genomic deletion, and/or *in vitro* conditions for virus propagation.

Overall, ORFV pathogenesis studies in the natural host using viruses lacking single NF-κB inhibitor genes have shown a remarkable spectrum of phenotypes, ranging from wild type disease (ORFV002, ORFV024) to moderate (ORFV073, ORFV119), or marked attenuation (ORFV121) [35–38]. The multiple NF-κB inhibitors encoded by a poxvirus together with the possibility of overlapping or complementing functions may explain these observations. Alternatively, specific poxviral NF-κB inhibitors may exert only subtle and perhaps transient host range effects on specific infected cells or the infected tissue microenvironment. Regardless, the impact of these subtle changes on viral fitness in nature may be difficult to fully ascertain under experimental conditions.

## Supporting information

S1 Fig Growth characteristics of deletion mutant virus OV-IA82-Δ119. OFTu cells were infected with wild type OV-IA82, deletion mutant OV-IA82-Δ119, or revertant OV-IA82-RV119^Flag^ viruses (MOI, 10), and titers were determined at 6 h, 12 h, 36 h, and 48 h p.i. and expressed as TCID50/ml. Results are mean values from two independent experiments.
(TIF)

S2 Fig Co-immunoprecipitation of ORFV119 with pRb. (A and B) HeLa cells were transfected with control plasmid (pFlag) or pORFV119^Flag^ and harvested at 12h post transfection. Total cell lysate and proteins extracts immunoprecipitated with antibodies against anti-Flag (A) or anti-pRb (B), were examined by Western blot (WB) using anti-pRb or anti-Flag antibodies. Results are representative of two independent experiments. Percentage of pRb co-immunoprecipitated by ORFV119^Flag^: 21.5±2.34%; Percentage of ORFV119^Flag^ co-immunoprecipitated by pRb: 38.7±2.61%.
(TIF)

S3 Fig Effect of ORFV119 on NF-κB-p65 activation during ORFV infection. (A) OFTu cells infected with OV-IA82 or OV-IA82-Δ119 (MOI, 10) were harvested at indicated times p.i. Total cell protein extracts (50 μg) were resolved by SDS-PAGE, blotted and probed with antibodies against total or Phospho (Ser536) NF-κB-p65. (B) Densitometry of Phospho NF-κB-p65 bands were normalized to the total NF-κB-p65 bands. Fold changes are shown relative to OV-IA82 treatment and results are mean values of two independent experiments (*P < 0.*05*).
(TIF)

S4 Fig Effect of ORFV119 on E2F-mediated luciferase activity in ORFV infected cells. OFTu cells were co-transfected with a pE2F-Luc and pRL-TK plasmids. At 24 h post transfection cells were mock infected or infected with OV-IA82, OV-IA82Δ119, or OV-IA82-RV119^LxGxE-Flag^. Firefly and sea pansy luciferase activities were measured at 1, 2, 4 and 6 h p.i. and expressed as relative fold changes in luciferase activity compared to mock treatment (*P<0.05; ** P<0.*01)*. Results are mean values of three independent experiments. No significant difference was observed between the viruses.
(TIF)

S5 Fig Effect of ORFV119 on poly(I:C), poly(A:T) or ORFV DNA induced NF-κB-mediated luciferase activity in HeLa cells. HeLa cells were co-transfected with pNF-κB-Luc, pRL-TK and pFlag or pORFV119^Flag^. At 24 h after transfection, cells were induced with poly(I:C) (500 ng), poly(A:T) (750 ng) or ORFV DNA (1 μg). Cells were harvested at 20 h p.i., and firefly and sea pansy luciferase activities were measured and expressed as fold changes in luciferase activity compared to uninduced (UN) cells (*P<0.*05)*. Results are mean values of two independent experiments.
(TIF)

S6 Fig Effect of OV-IA82-RV119^LxGxE-Flag^ or OV-IA82-RV119^LxGxE-Flag-Rb-^ virus in NF-κB-p65 nuclear translocation in OFTu or OFTu^Rb-^ cells. (A) OFTu or OFTu^Rb-^ cells were infected with OV-IA82-RV119^LxGxE-Flag^ (LxGxE virus) or OV-IA82-RV119^LxGxE-Flag-Rb-^ (LxGxE^Rb-^ virus) as described in Materials and Methods and cells were fixed at 1 h p.i. sequentially probed with antibody against NF-κB-p65 and Alexa Fluor 594 labeled secondary antibody, counterstained with DAPI, and examined by confocal microscopy. Red, NF-κB-p65; Blue, DAPI. (B) Cells were counted (n = 500 cells/slide) and results are shown as percentage of cells expressing nuclear NF-κB-p65. Results are mean values from two independent experiments. *p* values for LxGxE virus/OFTu cells vs LxGxE virus/OFTu^Rb-^ cells, LxGxE^Rb-^ virus/OFTu cells and LxGxE^Rb-^ virus/OFTu^Rb-^ cells were not significant (*P>0*.*05*).
(TIF)

S7 Fig Co-immunoprecipitation of ORFV119^LxGxE^ with TRAF2. (A and B) 293T cells were transfected with control plasmid (pFlag) or pORFV119^LxGxE-Flag^ (p119^LxGxE-Flag^) and harvested at 12h post transfection. Total cell lysate and proteins extracts immunoprecipitated with antibodies against anti-Flag (A) or anti-TRAF2 (B), were examined by Western blot (WB) using anti-TRAF2 or anti-Flag antibodies. Results are representative of two independent experiments.
(TIF)

S8 Fig Effect of ORFV119 on ORFV virulence in the natural host. Sheep were topically inoculated with OV-IA82-Δ119 (sheep # 39, 43, 513, and 622), OV-IA82-RV119^Flag^ (sheep # 19, 79, 101 and 639) (0.5 ml, 10^7^ TCID_50_/ml) or PBS (3 animals) on the scarified skin of the right lower lip. (A). Clinical course of disease for OV-IA82-Δ119 (Δ119) and OV-IA82-RV119^Flag^ (RV119). Results are shown for 5, 9, 16, and 21 days p.i. (B). Comparison of mean lesion length (in cm) at indicated time points.
(TIF)

## References

[1] Fleming SB, Wise LM, Mercer AA. Molecular genetic analysis of orf virus: A poxvirus that has adapted to skin. Viruses. 2015;7: 1505–1539. doi: 10.3390/v7031505 25807056 10.3390/v7031505 PMC4379583

[2] Haig DM, Mercer AA. Ovine diseases. Orf. Vet. Res. 1998;29: 311–326. 9689744

[3] Erickson GA, Carbrey EA, Gustafson GA. Generalized contagious ecthyma in a sheep rancher: diagnostic considerations. J Am Vet Med Assoc. 1975;166: 262–263. 1094013

[4] Meechan JG, MacLeod RI. Human labial orf: a case report. Br Dent J. 1992; 173: 343–344. 1467011 10.1038/sj.bdj.4808050

[5] Sanchez RL, Hebert A, Lucia H, Swedo J. Orf. A case report with histologic, electron microscopic, and immunoperoxidase studies. Arch Pathol Lab Med. 1985;109: 166–170. 3883947

[6] McKeever DJ, Jenkinson DM, Hutchison G, Reid HW. Studies of the pathogenesis of orf virus infection in sheep. J Comp Pathol. 1988;99: 317–328. 3204166 10.1016/0021-9975(88)90052-7

[7] Kalali BN, Köllisch G, Mages J, Müller T, Bauer S, Wagner H, Ring J, Lang R, Mempel M, Ollert M. Double-stranded RNA induces an antiviral defense status in epidermal keratinocytes through TLR3-, PKR-, and MDA5/RIG-I-mediated differential signaling. J Immunol. 2008;181: 2694–2704. 18684960 10.4049/jimmunol.181.4.2694

[8] Nestle FO, Di Meglio P, Qin JZ, Nickoloff BJ. Skin immune sentinels in health and disease. Nat Rev Immunol. 2009;9: 679–691. doi: 10.1038/nri2622 19763149 10.1038/nri2622 PMC2947825

[9] Kaufman CK, Fuchs E. It's got you covered: NF-kappaB in the epidermis. J Cell Biol. 2000;149: 999–1004. 10831603 10.1083/jcb.149.5.999 PMC2174820

[10] Vallabhapurapu S, Karin M. Regulation and function of NFkappaB transcription factors in the immune system. Annu Rev Immunol. 2009;27: 693–733. doi: 10.1146/annurev.immunol.021908.132641 19302050 10.1146/annurev.immunol.021908.132641

[11] Scheidereit C. IkappaB kinase complexes: gateways to NF-kappaB activation and transcription. Oncogene. 2006;25: 6685–6705. doi: 10.1038/sj.onc.1209934 17072322 10.1038/sj.onc.1209934

[12] Chen L-F, Greene WC. Shaping the nuclear action of NF-kappaB. Nat Rev Mol Cell Biol. 2004;5: 392–401. doi: 10.1038/nrm1368 15122352 10.1038/nrm1368

[13] Amaya M, Keck F, Bailey C, Narayanan A. The role of the IKK complex in viral infections. Pathog Dis. 2014;72: 32–44. doi: 10.1111/2049-632X.12210 25082354 10.1111/2049-632X.12210 PMC7108545

[14] Hayden MS, Ghosh S. Shared principles in NF-kappaB signaling. Cell. 2008;132: 344–362. doi: 10.1016/j.cell.2008.01.020 18267068 10.1016/j.cell.2008.01.020

[15] Chen ZJ. Ubiquitin signaling in the NF-kappaB pathway. Nat Cell Biol. 2005;7: 758–765. doi: 10.1038/ncb0805-758 16056267 10.1038/ncb0805-758 PMC1551980

[16] Adhikari A, Xu M, Chen ZJ. Ubiquitin-mediated activation of TAK1 and IKK. Oncogene. 2007;26: 3214–3226. doi: 10.1038/sj.onc.1210413 17496917 10.1038/sj.onc.1210413

[17] Wang C, Deng L, Hong M, Akkaraju GR, Inoue J, Chen ZJ. TAK1 is a ubiquitin-dependent kinase of MKK and IKK. Nature. 2001;412: 346–351. doi: 10.1038/35085597 11460167 10.1038/35085597

[18] Deng L, Wang C, Spencer E, Yang L, Braun A, You J, Slaughter C, Pickart C, Chen ZJ. Activation of the IkappaB kinase complex by TRAF6 requires a dimeric ubiquitin-conjugating enzyme complex and a unique polyubiquitin chain. Cell. 2000;103: 351–361. 11057907 10.1016/s0092-8674(00)00126-4

[19] Brady G, Bowie AG. Innate immune activation of NFkappaB and its antagonism by poxviruses. Cytokine Growth Factor Rev. 2014;25: 611–620. doi: 10.1016/j.cytogfr.2014.07.004 25081317 10.1016/j.cytogfr.2014.07.004

[20] Smith GL, Benfield CT, Maluquer de Motes C, Mazzon M, Ember SW, Ferguson BJ, Sumner RP. Vaccinia virus immune evasion: mechanisms, virulence and immunogenicity. J Gen Virol. 2013;94: 2367–2392. doi: 10.1099/vir.0.055921-0 23999164 10.1099/vir.0.055921-0

[21] Bowie A, Kiss-Toth E, Symons JA, Smith GL, Dower SK, O’Neill LA. A46R and A52R from vaccinia virus are antagonists of host IL-1 and toll-like receptor signaling. Proc Natl Acad Sci U S A. 2000;97: 10162–10167. doi: 10.1073/pnas.160027697 10920188 10.1073/pnas.160027697 PMC27775

[22] Chen RA-J, Ryzhakov G, Cooray S, Randow F, Smith GL. Inhibition of IkappaB kinase by vaccinia virus virulence factor B14. PLoS Pathog. 2008;4: e22 doi: 10.1371/journal.ppat.0040022 18266467 10.1371/journal.ppat.0040022 PMC2233672

[23] Ember SWJ, Ren H, Ferguson BJ, Smith GL. Vaccinia virus protein C4 inhibits NF-κB activation and promotes virus virulence. J Gen Virol. 2012;93: 2098–2108. doi: 10.1099/vir.0.045070-0 22791606 10.1099/vir.0.045070-0 PMC3541790

[24] DiPerna G, Stack J, Bowie AG, Boyd A, Kotwal G, Zhang Z, Arvikar S, Latz E, Fitzgerald KA, Marshall WL. Poxvirus protein N1L targets the I-kappaB kinase complex, inhibits signaling to NF-kappaB by the tumor necrosis factor superfamily of receptors, and inhibits NF-kappaB and IRF3 signaling by toll-like receptors. J Biol Chem. 2004;279: 36570–36578. doi: 10.1074/jbc.M400567200 15215253 10.1074/jbc.M400567200

[25] Gedey R, Jin X-L, Hinthong O, Shisler JL. Poxviral regulation of the host NF-kappaB response: the vaccinia virus M2L protein inhibits induction of NF-kappaB activation via an ERK2 pathway in virus-infected human embryonic kidney cells. J Virol. 2006;80: 8676–8685. doi: 10.1128/JVI.00935-06 16912315 10.1128/JVI.00935-06 PMC1563854

[26] Shisler JL, Jin X-L. The vaccinia virus K1L gene product inhibits host NF-kappaB activation by preventing IkappaBalpha degradation. J Virol. 2004;78: 3553–3560. doi: 10.1128/JVI.78.7.3553-3560.2004 15016878 10.1128/JVI.78.7.3553-3560.2004 PMC371086

[27] Mansur DS, Maluquer de Motes C, Unterholzner L, Sumner RP, Ferguson BJ, Ren H, Strnadova P, Bowie AG, Smith GL. Poxvirus targeting of E3 ligase β-TrCP by molecular mimicry: a mechanism to inhibit NF-κB activation and promote immune evasion and virulence. PLoS Pathog. 2013;9: e1003183 doi: 10.1371/journal.ppat.1003183 23468625 10.1371/journal.ppat.1003183 PMC3585151

[28] Myskiw C, Arsenio J, van Bruggen R, Deschambault Y, Cao J. Vaccinia virus E3 suppresses expression of diverse cytokines through inhibition of the PKR, NF-kappaB, and IRF3 pathways. J Virol. 2009;83: 6757–6768. doi: 10.1128/JVI.02570-08 19369349 10.1128/JVI.02570-08 PMC2698532

[29] Schröder M, Baran M, Bowie AG. Viral targeting of DEAD box protein 3 reveals its role in TBK1/IKKepsilon-mediated IRF activation. EMBO J. 2008;27: 2147–2157. doi: 10.1038/emboj.2008.143 18636090 10.1038/emboj.2008.143 PMC2516890

[30] Bartlett N, Symons JA, Tscharke DC, Smith GL. The vaccinia virus N1L protein is an intracellular homodimer that promotes virulence. J Gen Virol. 2002;83: 1965–1976. doi: 10.1099/0022-1317-83-8-1965 12124460 10.1099/0022-1317-83-8-1965

[31] Camus-Bouclainville C, Fiette L, Bouchiha S, Pignolet B, Counor D, Filipe C, Gelfi J, Messud-Petit F. A virulence factor of myxoma virus colocalizes with NF-kappaB in the nucleus and interferes with inflammation. J Virol. 2004;78: 2510–2516. doi: 10.1128/JVI.78.5.2510-2516.2004 14963153 10.1128/JVI.78.5.2510-2516.2004 PMC369233

[32] Chen RA, Jacobs N, Smith GL. Vaccinia virus strain Western Reserve protein B14 is an intracellular virulence factor. J Gen Virol. 2006;87: 1451–1458. doi: 10.1099/vir.0.81736-0 16690909 10.1099/vir.0.81736-0

[33] Harte MT, Haga IR, Maloney G, Gray P, Reading PC, Bartlett NW, Smith GL, Bowie A, O'Neill LA. The poxvirus protein A52R targets Toll-like receptor signaling complexes to suppress host defense. J Exp Med. 2003;197: 343–351. doi: 10.1084/jem.20021652 12566418 10.1084/jem.20021652 PMC2193841

[34] Mohamed MR, Rahman MM, Rice A, Moyer RW, Werden SJ, McFadden G. Cowpox virus expresses a novel ankyrin repeat NFkappaB inhibitor that controls inflammatory cell influx into virus-infected tissues and is critical for virus pathogenesis. J Virol. 2009;83: 9223–9236. doi: 10.1128/JVI.00861-09 19570875 10.1128/JVI.00861-09 PMC2738262

[35] Diel DG, Delhon G, Luo S, Flores EF, Rock DL. A novel inhibitor of the NF- {kappa}B signaling pathway encoded by the parapoxvirus orf virus. J Virol. 2010;84: 3962–3973. doi: 10.1128/JVI.02291-09 20147406 10.1128/JVI.02291-09 PMC2849485

[36] Diel DG, Luo S, Delhon G, Peng Y, Flores EF, Rock DL. A nuclear inhibitor of NF-kappaB encoded by a poxvirus. J Virol. 2011;85: 264–275. doi: 10.1128/JVI.01149-10 20980501 10.1128/JVI.01149-10 PMC3014193

[37] Diel DG, Luo S, Delhon G, Peng Y, Flores EF, Rock DL. Orf virus ORFV121 encodes a novel inhibitor of NF-kappaB that contributes to virus virulence. J Virol. 2011;85: 2037–2049. doi: 10.1128/JVI.02236-10 21177808 10.1128/JVI.02236-10 PMC3067802

[38] Khatiwada S, Delhon G, Nagendraprabhu P, Chaulagain S, Luo S, Diel DG, Flores EF, Rock DL. A parapoxviral virion protein inhibits NF-κB signaling early in infection. PLoS Pathog. 2017;13: e1006561 doi: 10.1371/journal.ppat.1006561 28787456 10.1371/journal.ppat.1006561 PMC5560748

[39] Ning Z, Zheng Z, Hao W, Duan C, Li W, Wang Y, Li M, Luo S. The N terminus of orf virus-encoded protein 002 inhibits acetylation of NF-κB p65 by preventing Ser(276) phosphorylation. PLoS One. 2013;8: e58854 doi: 10.1371/journal.pone.0058854 23536830 10.1371/journal.pone.0058854 PMC3594181

[40] Dick FA, Rubin SM. Molecular mechanisms underlying RB protein function. Nat Rev Mol Cell Biol. 2013;14: 297–306. doi: 10.1038/nrm3567 23594950 10.1038/nrm3567 PMC4754300

[41] Ferrari R, Gou D, Jawdekar G, Johnson SA, Nava M, Su T, Yousef AF, Zemke NR, Pellegrini M, Kurdistani SK, Berk AJ. Adenovirus Small E1A Employs the Lysine Acetylases p300/CBP and Tumor Suppressor Rb to Repress Select Host Genes and Promote Productive Virus Infection. Cell Host Microbe. 2014;16: 663–676. doi: 10.1016/j.chom.2014.10.004 25525796 10.1016/j.chom.2014.10.004 PMC4418520

[42] Kalejta RF, Bechtel JT, Shenk T. Human cytomegalovirus pp71 stimulates cell cycle progression by inducing the proteasome-dependent degradation of the retinoblastoma family of tumor suppressors. Mol Cell Biol. 2003;23: 1885–1895. doi: 10.1128/MCB.23.6.1885-1895.2003 12612064 10.1128/MCB.23.6.1885-1895.2003 PMC149485

[43] Gonzalez SL, Stremlau M, He X, Basile JR, Munger K. Degradation of the retinoblastoma tumor suppressor by the human papillomavirus type 16 E7 oncoprotein is important for functional inactivation and is separable from proteasomal degradation of E7. J Virol. 2001;75: 7583–7591. doi: 10.1128/JVI.75.16.7583-7591.2001 11462030 10.1128/JVI.75.16.7583-7591.2001 PMC114993

[44] Cook JL, Walker TA, Worthen GS, Radke JR. Role of the E1A Rb-binding domain in repression of the NF-kappa B-dependent defense against tumor necrosis factor-alpha. Proc Natl Acad Sci U S A. 2002;99: 9966–9971. doi: 10.1073/pnas.162082999 12119420 10.1073/pnas.162082999 PMC126608

[45] Garcia MA, Gallego P, Campagna M, González-Santamaría J, Martínez G, Marcos-Villar L, Vidal A, Esteban M, Rivas C. Activation of NF-κB Pathway by Virus Infection Requires Rb Expression. PLoS One. 2009;4: e6422 doi: 10.1371/journal.pone.0006422 19649275 10.1371/journal.pone.0006422 PMC2713421

[46] Singh M, Krajewski M, Mikolajka A, Holak TA. Molecular Determinants for the Complex Formation between the Retinoblastoma Protein and LXCXE Sequences. J Biol Chem. 2005;280: 37868–378676. doi: 10.1074/jbc.M504877200 16118215 10.1074/jbc.M504877200

[47] Delhon G, Tulman ER, Afonso CL, Lu Z, de la Concha-Bermejillo A, Lehmkuhl HD, Piccone ME, Kutish GF, Rock DL. Genomes of the parapoxviruses ORF virus and bovine papular stomatitis virus. J Virol. 2004;78: 168–177. doi: 10.1128/JVI.78.1.168-177.2004 14671098 10.1128/JVI.78.1.168-177.2004 PMC303426

[48] Krauss O, Hollinshead R, Hollinshead M, Smith GL. An investigation of incorporation of cellular antigens into vaccinia virus particles. J Gen Virol. 2002;83: 2347–2359. doi: 10.1099/0022-1317-83-10-2347 12237415 10.1099/0022-1317-83-10-2347

[49] Wang X, Zhang J, Hao W, Peng Y, Li H, Li W, Li M, Luo S. Isolation and Characterization of Monoclonal Antibodies Against a Virion Core Protein of Orf Virus Strain NA1/11 As Potential Diagnostic Tool for Orf Viruses. Monoclon Antib Immunodiagn Immunother. 2015;34: 233–245. doi: 10.1089/mab.2014.0101 26301926 10.1089/mab.2014.0101 PMC4556089

[50] Li M, Zhang Z, Koh H, Lu R, Jiang Z, Alioua A, Garcia-Valdes J, Stefani E, Toro L. The β1-subunit of the MaxiK channel associates with the thromboxane A2 receptor and reduces thromboxane A2 functional effects. J Biol Chem. 2013;288: 3668–3677. doi: 10.1074/jbc.M112.426585 23255603 10.1074/jbc.M112.426585 PMC3561584

[51] Friederichs S, Krebs S, Blum H, Lang H, Büttner M. Parapoxvirus (PPV) of red deer reveals subclinical infection and confirms a unique species. J Gen Virol. 2015;96: 1446–1462. doi: 10.1099/vir.0.000080 25701822 10.1099/vir.0.000080

[52] Harbour JW, Dean DC. Rb function in cell-cycle regulation and apoptosis. Nat Cell Biol. 2000;2: E65–E67. doi: 10.1038/35008695 10783254 10.1038/35008695

[53] Balassu TC, Robinson AJ. Orf virus replication in bovine testis cells: kinetics of viral DNA, polypeptide, and infectious virus production and analysis of virion polypeptides. Arch Virol. 1987;97: 267–281. 3426396 10.1007/BF01314426

[54] Dowdy SF, Hinds PW, Louie K, Reed SI, Arnold A, Weinberg RA. Physical Interaction of the Retinoblastoma Protein with Human D Cyclins. Cell. 1993;73: 499–511. 8490963 10.1016/0092-8674(93)90137-f

[55] Shew JY, Lin BT, Chen PL, Tseng BY, Yang-Feng TL, Lee WH. C-terminal truncation of the retinoblastoma gene product leads to functional inactivation. Proc Natl Acad Sci U S A.1990;87: 6–10. 1688660 10.1073/pnas.87.1.6 PMC53188

[56] Blanpain C, Fuchs E. Epidermal homeostasis: a balancing act of stem cells in the skin. Nat Rev Mol Cell Biol. 2009;10: 207–217. doi: 10.1038/nrm2636 19209183 10.1038/nrm2636 PMC2760218

[57] Pasparakis M. Regulation of tissue homeostasis by NF-kappaB signaling: implications for inflammatory diseases. Nat Rev Immunol. 2009;9: 778–788. doi: 10.1038/nri2655 19855404 10.1038/nri2655

[58] Wullaert A, Bonnet MC, Pasparakis M. NF-κB in the regulation of epithelial homeostasis and inflammation. Cell Res. 2011;21: 146–158. doi: 10.1038/cr.2010.175 21151201 10.1038/cr.2010.175 PMC3193399

[59] Mohr S, Grandemange S, Massimi P, Darai G, Banks L, Martinou JC, Zeier M, Muranyi W. Targeting the Retinoblastoma Protein by MC007L, Gene Product of the Molluscum Contagiosum Virus: Detection of a Novel Virus-Cell Interaction by a Member of the Poxviruses. J Virol. 2008;82: 10625–10633. doi: 10.1128/JVI.01187-08 18701596 10.1128/JVI.01187-08 PMC2573180

[60] Borghi A, Verstrepen L, Beyaert R. TRAF2 multitasking in TNF receptor-induced signaling to NF-κB, MAP kinases and cell death. Biochem Pharmacol. 2016;116: 1–10. doi: 10.1016/j.bcp.2016.03.009 26993379 10.1016/j.bcp.2016.03.009

[61] Randall CM, Jokela JA, Shisler JL. The MC159 protein from the molluscum contagiosum poxvirus inhibits NF-κB activation by interacting with the IκB kinase complex. J Immunol. 2012;188: 2371–2379. doi: 10.4049/jimmunol.1100136 22301546 10.4049/jimmunol.1100136 PMC3288875

[62] Guasparri I, Wu H, Cesarman E. The KSHV oncoprotein vFLIPcontains a TRAF‐interacting motif and requires TRAF2 and TRAF3 for signaling. EMBO rep. 2006;7: 114–119. doi: 10.1038/sj.embor.7400580 16311516 10.1038/sj.embor.7400580 PMC1369231

[63] LaMonica R, Kocer SS, Nazarova J, Dowling W, Geimonen E, Shaw RD, Mackow ER. VP4 differentially regulates TRAF2 signaling, disengaging JNK activation while directing NF-κB to effect rotavirus-specific cellular responses. J Biol Chem. 2001;276: 19889–19896. doi: 10.1074/jbc.M100499200 11262403 10.1074/jbc.M100499200

[64] Haga IR, Jowers TP, Griffiths SJ, Haas J, Beard PM. TRAF2 facilitates vaccinia virus replication by promoting rapid virus entry. J Virol. 2014;88: 3664–3677. doi: 10.1128/JVI.03013-13 24429366 10.1128/JVI.03013-13 PMC3993545

[65] Laliberte JP, Weisberg AS, Moss B. The membrane fusion step of vaccinia virus entry is cooperatively mediated by multiple viral proteins and host cell components. PLoS Pathog. 2011;7: e1002446 doi: 10.1371/journal.ppat.1002446 22194690 10.1371/journal.ppat.1002446 PMC3240603

[66] Bravo Cruz AG, Shisler JL. Vaccinia virus K1 ankyrin repeat protein inhibits NF-κB activation by preventing RelA acetylation. J Gen Virol. 2016;97: 2691–2702. doi: 10.1099/jgv.0.000576 27503790 10.1099/jgv.0.000576

[67] Hautaniemi M, Ueda N, Tuimala J, Mercer AA, Lahdenperä J, McInnes CJ. The genome of pseudocowpoxvirus: comparison of a reindeer isolate and a reference strain. J Gen Virol. 2010;91: 1560–1576. doi: 10.1099/vir.0.018374-0 20107016 10.1099/vir.0.018374-0

[68] Hautaniemi M, Vaccari F, Scacliarini A, Laaksonen S, Huovilainen A, McInnes CJ. Analysis of deletion within the reindeer pseudocowpoxvirus genome. Virus Res. 2011;160: 326–332. doi: 10.1016/j.virusres.2011.07.005 21798294 10.1016/j.virusres.2011.07.005

[69] Chi X, Zeng X, Li W, Hao W, Li M, Huang X, Huang Y, Rock DL, Luo S, Wang S. Genome analysis of orf virus isolates from goats in the Fujian Province of southern China. Front Microbiol. 2015;6: 1135 doi: 10.3389/fmicb.2015.01135 26557108 10.3389/fmicb.2015.01135 PMC4616995

[70] Huang T, Tulman ER, Diel DG, Khatiwada S, Sims W, Edwards JF, Wen X, Kutish GF, Rock DL, Delhon G. Coinfection with multiple strains of Bovine Papular Stomatitis Virus. Arch Virol. 2015;160: 1527–1532. doi: 10.1007/s00705-015-2394-2 25804193 10.1007/s00705-015-2394-2

[71] Qiao J, Yang HB, Peng YL, Meng QL, Chen C, Ma Y, Xie K, Liu TL, Cai XP, Chen CF. Effect of ORF119 gene deletion on the replication and virulence of orf virus. Acta Virologica. 2015;59: 257–264. 26435149 10.4149/av_2015_03_257

