# Supplementary figures and images for: A parapoxviral virion protein targets the retinoblastoma protein to inhibit NF-κB signaling
Source: PLoS Pathog. 2017 Dec 15;13(12):e1006779. doi: 10.1371/journal.ppat.1006779 (PMC5747488; doi:10.1371/journal.ppat.1006779)

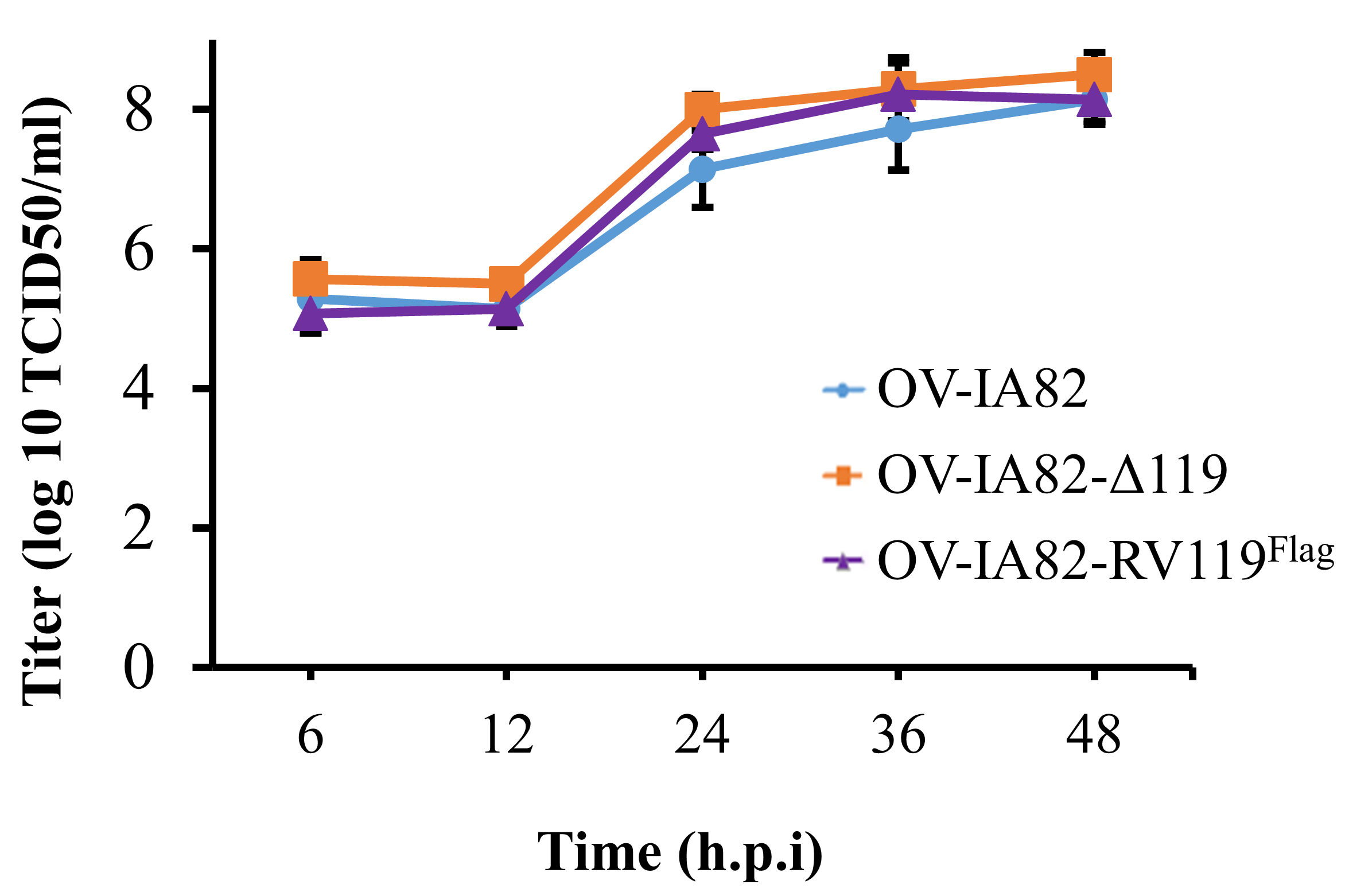

Supplement: S1 Fig — OFTu cells were infected with wild type OV-IA82, deletion mutant OV-IA82-Δ119, or revertant OV-IA82-RV119Flag viruses (MOI, 10), and titers were determined at 6 h, 12 h, 36 h, and 48 h p.i. and expressed as TCID50/ml. Results are mean values from two independent experiments. (TIF) [file ppat.1006779.s001.tif]

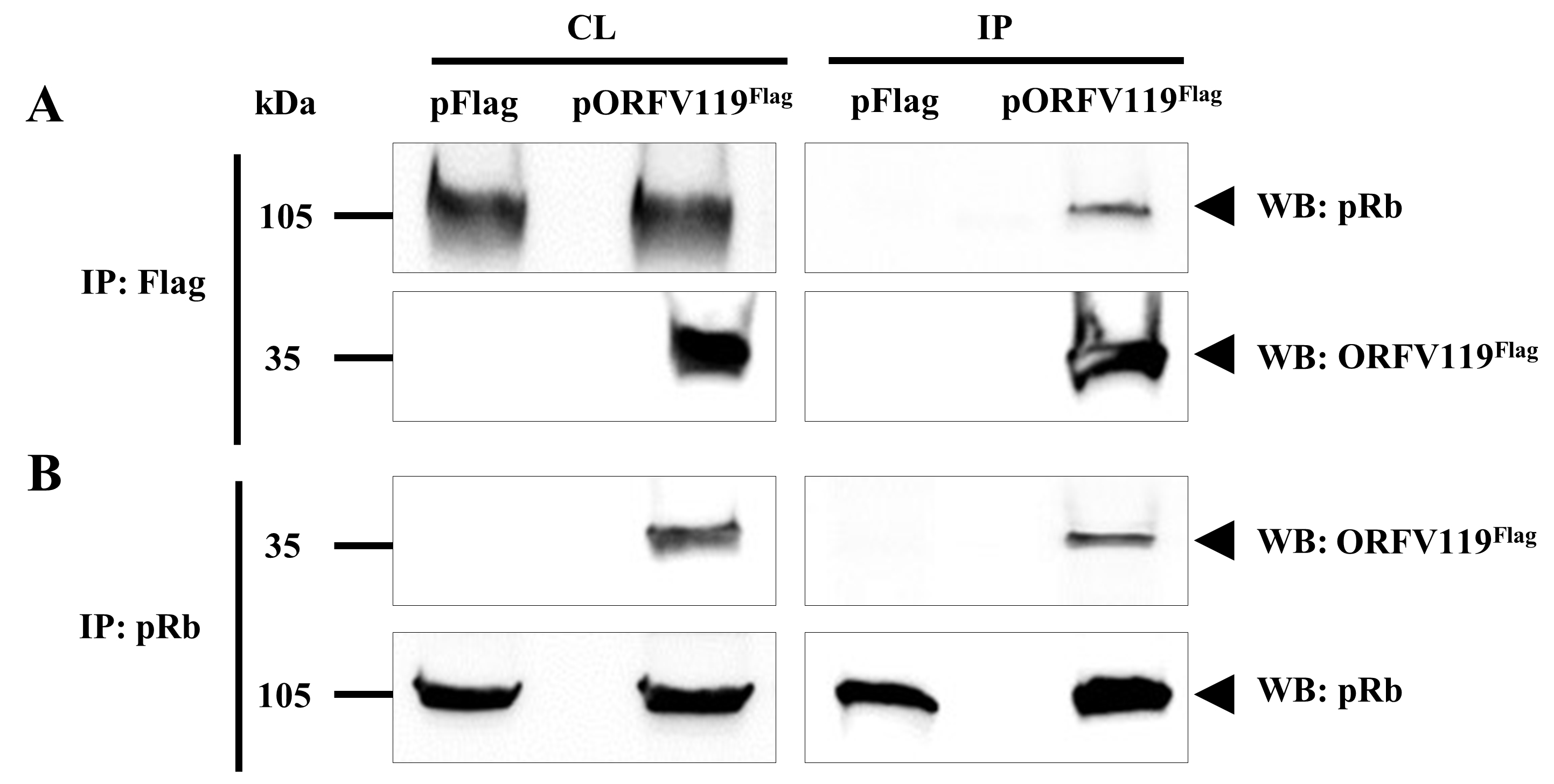

Supplement: S2 Fig — (A and B) HeLa cells were transfected with control plasmid (pFlag) or pORFV119Flag and harvested at 12h post transfection. Total cell lysate and proteins extracts immunoprecipitated with antibodies against anti-Flag (A) or anti-pRb (B), were examined by Western blot (WB) using anti-pRb or anti-Flag antibodies. Results are representative of two independent experiments. Percentage of pRb co-immunoprecipitated by ORFV119Flag: 21.5±2.34%; Percentage of ORFV119Flag co-immunoprecipitated by pRb: 38.7±2.61%. (TIF) [file ppat.1006779.s002.tif]

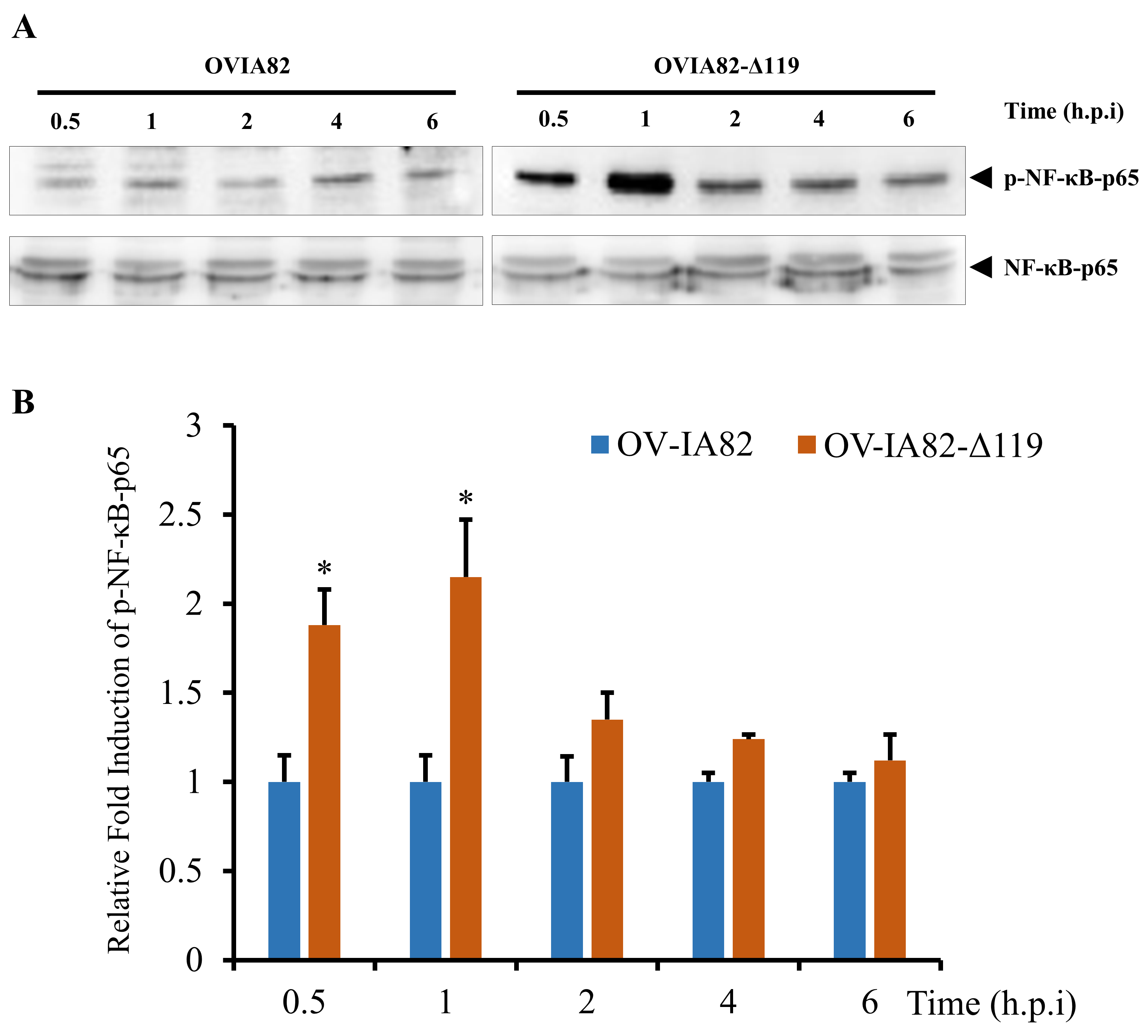

Supplement: S3 Fig — (A) OFTu cells infected with OV-IA82 or OV-IA82-Δ119 (MOI, 10) were harvested at indicated times p.i. Total cell protein extracts (50 μg) were resolved by SDS-PAGE, blotted and probed with antibodies against total or Phospho (Ser536) NF-κB-p65. (B) Densitometry of Phospho NF-κB-p65 bands were normalized to the total NF-κB-p65 bands. Fold changes are shown relative to OV-IA82 treatment and results are mean values of two independent experiments (*P < 0.05). (TIF) [file ppat.1006779.s003.tif]

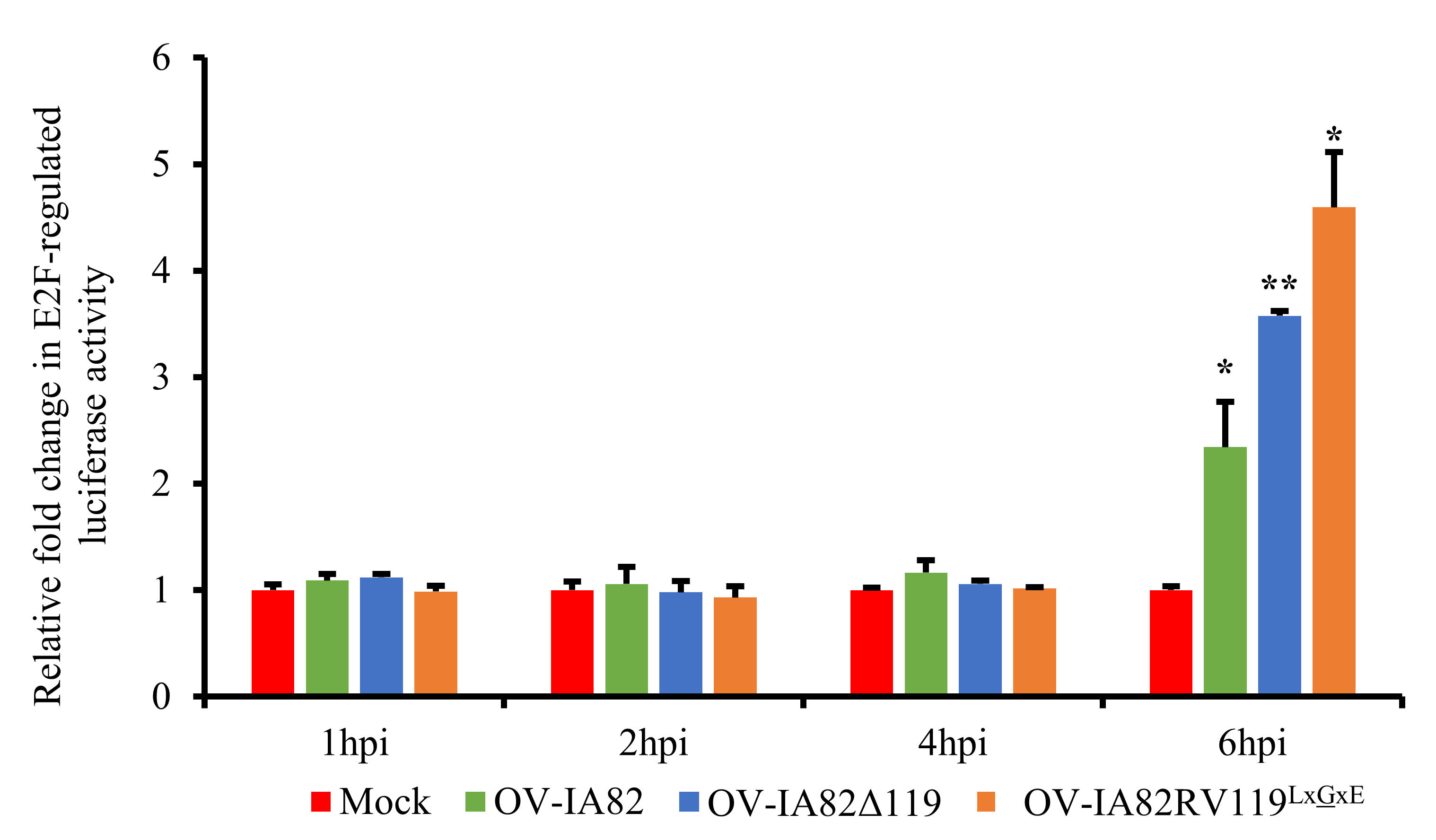

Supplement: S4 Fig — OFTu cells were co-transfected with a pE2F-Luc and pRL-TK plasmids. At 24 h post transfection cells were mock infected or infected with OV-IA82, OV-IA82Δ119, or OV-IA82-RV119LxGxE-Flag. Firefly and sea pansy luciferase activities were measured at 1, 2, 4 and 6 h p.i. and expressed as relative fold changes in luciferase activity compared to mock treatment (*P<0.05; ** P<0.01). Results are mean values of three independent experiments. No significant difference was observed between the viruses. (TIF) [file ppat.1006779.s004.tif]

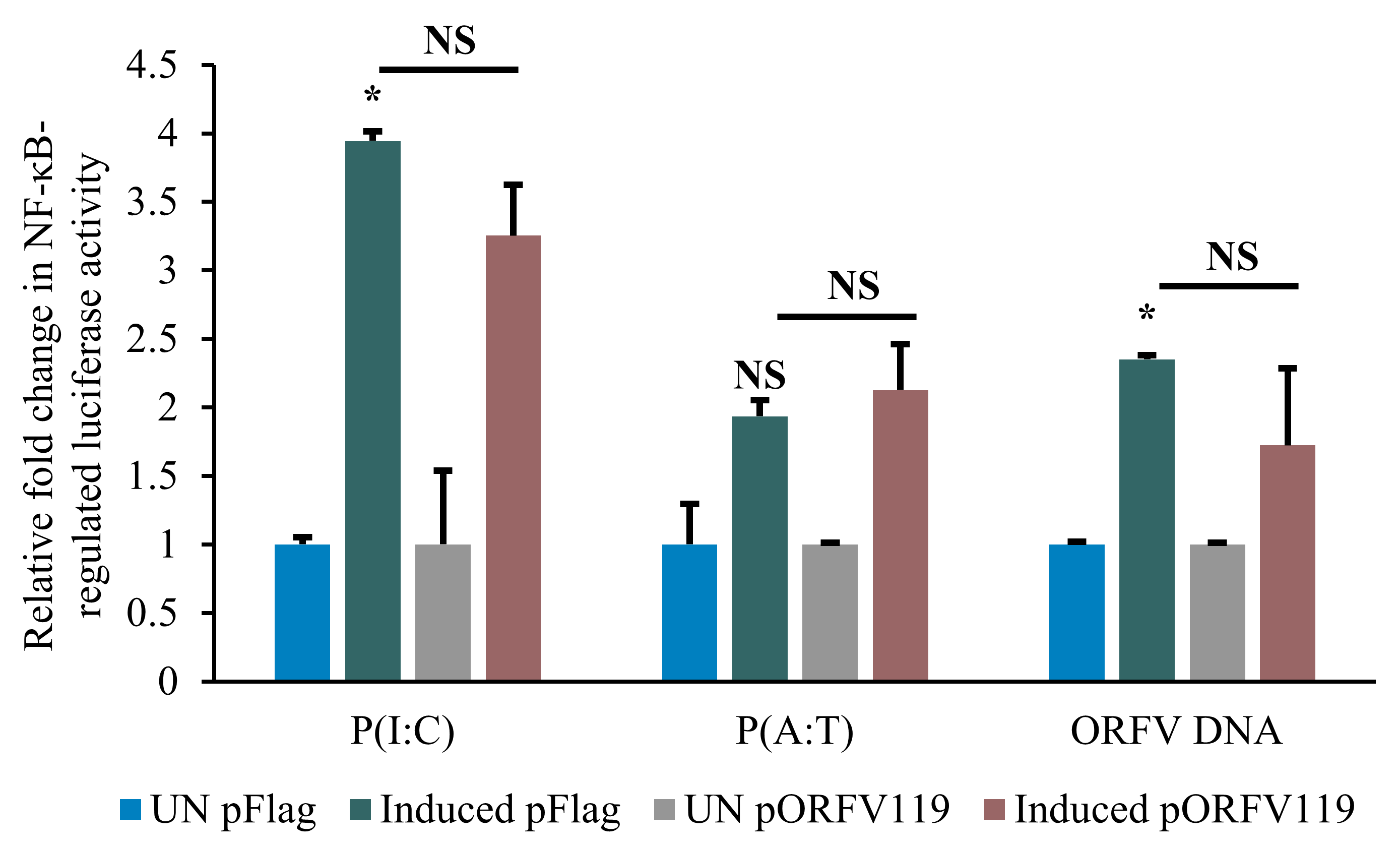

Supplement: S5 Fig — HeLa cells were co-transfected with pNF-κB-Luc, pRL-TK and pFlag or pORFV119Flag. At 24 h after transfection, cells were induced with poly(I:C) (500 ng), poly(A:T) (750 ng) or ORFV DNA (1 μg). Cells were harvested at 20 h p.i., and firefly and sea pansy luciferase activities were measured and expressed as fold changes in luciferase activity compared to uninduced (UN) cells (*P<0.05). Results are mean values of two independent experiments. (TIF) [file ppat.1006779.s005.tif]

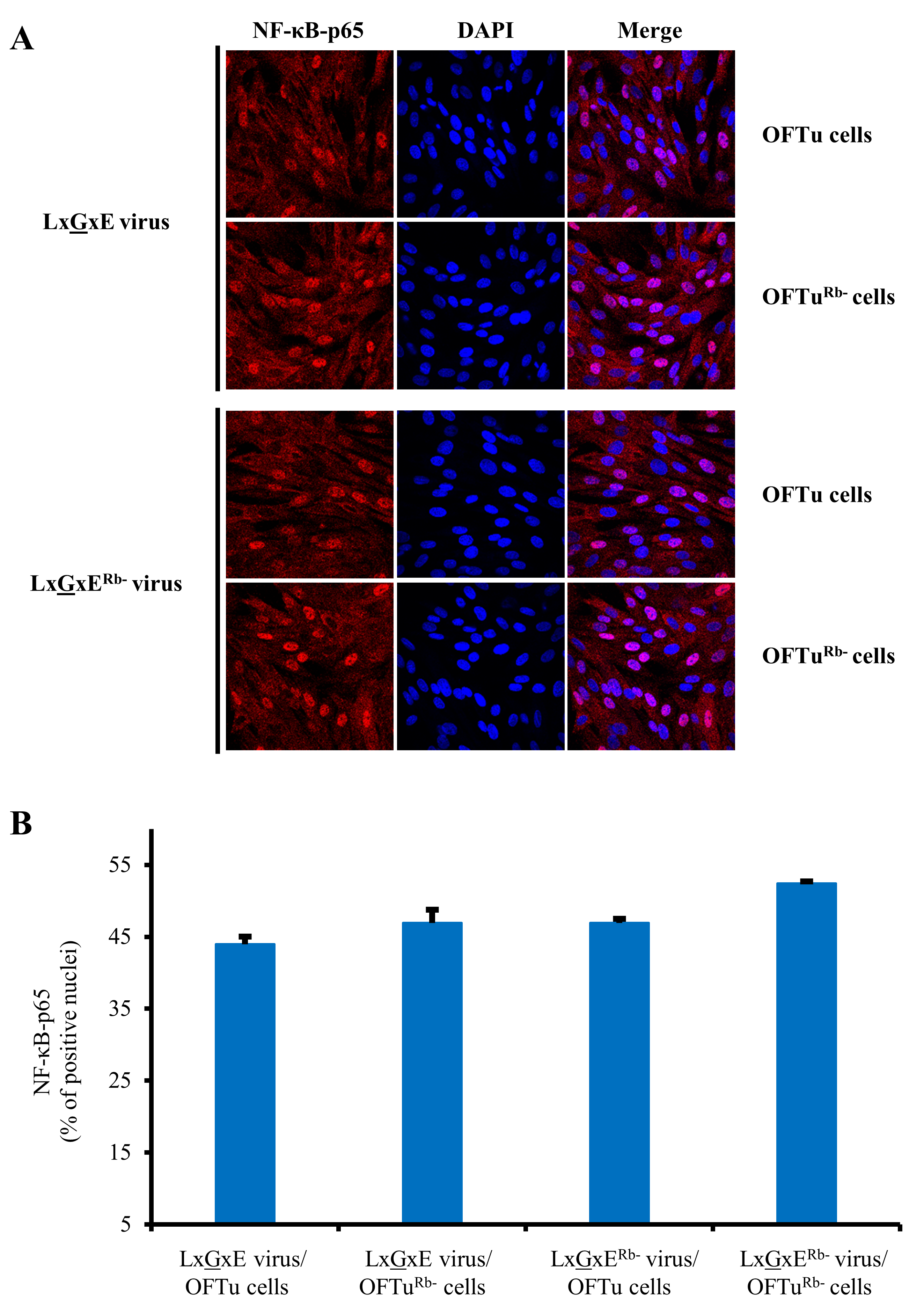

Supplement: S6 Fig — (A) OFTu or OFTuRb- cells were infected with OV-IA82-RV119LxGxE-Flag (LxGxE virus) or OV-IA82-RV119LxGxE-Flag-Rb- (LxGxERb- virus) as described in Materials and Methods and cells were fixed at 1 h p.i. sequentially probed with antibody against NF-κB-p65 and Alexa Fluor 594 labeled secondary antibody, counterstained with DAPI, and examined by confocal microscopy. Red, NF-κB-p65; Blue, DAPI. (B) Cells were counted (n = 500 cells/slide) and results are shown as percentage of cells expressing nuclear NF-κB-p65. Results are mean values from two independent experiments. p values for LxGxE virus/OFTu cells vs LxGxE virus/OFTuRb- cells, LxGxERb- virus/OFTu cells and LxGxERb- virus/OFTuRb- cells were not significant (P>0.05). (TIF) [file ppat.1006779.s006.tif]

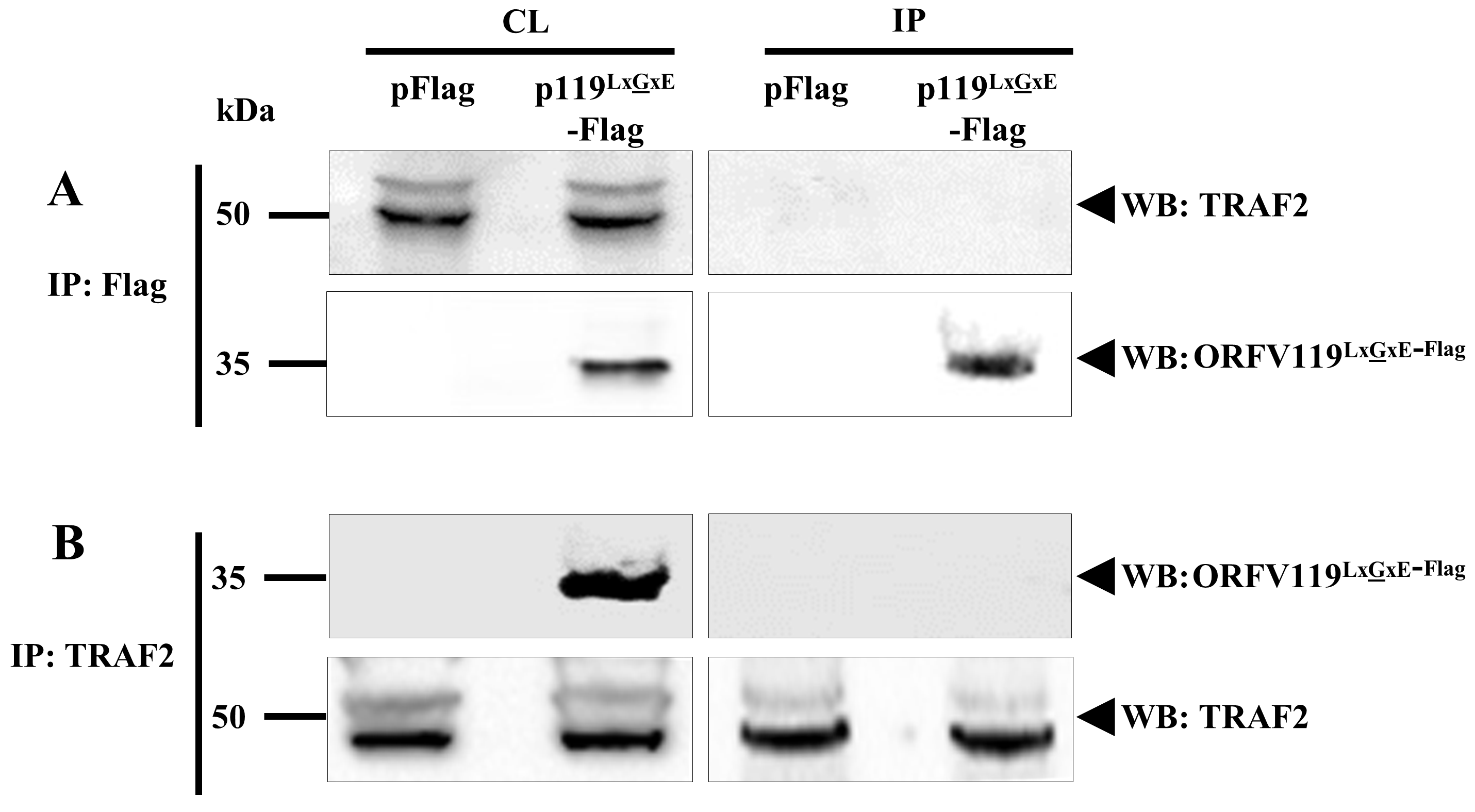

Supplement: S7 Fig — (A and B) 293T cells were transfected with control plasmid (pFlag) or pORFV119LxGxE-Flag (p119LxGxE-Flag) and harvested at 12h post transfection. Total cell lysate and proteins extracts immunoprecipitated with antibodies against anti-Flag (A) or anti-TRAF2 (B), were examined by Western blot (WB) using anti-TRAF2 or anti-Flag antibodies. Results are representative of two independent experiments. (TIF) [file ppat.1006779.s007.tif]

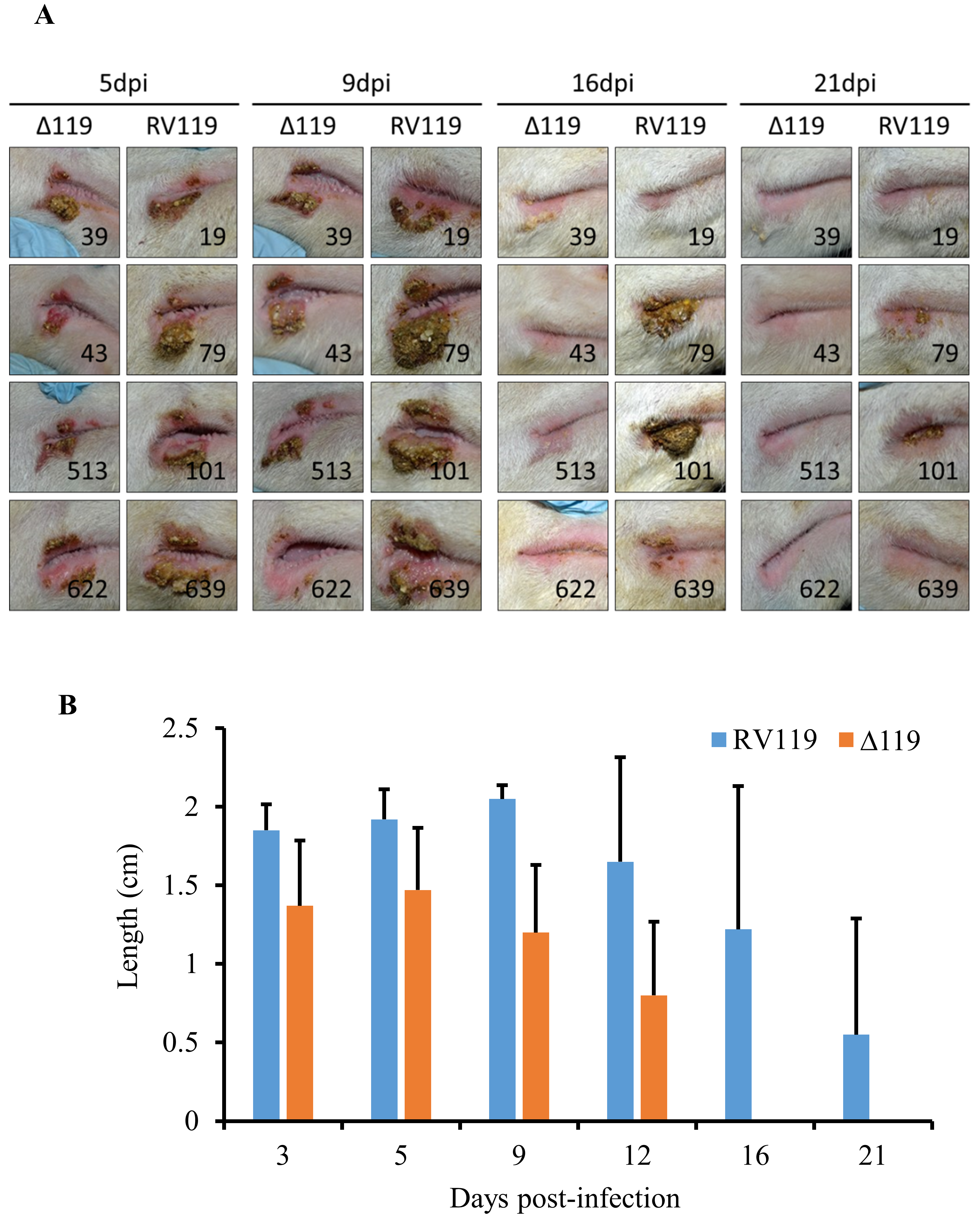

Supplement: S8 Fig — Sheep were topically inoculated with OV-IA82-Δ119 (sheep # 39, 43, 513, and 622), OV-IA82-RV119Flag (sheep # 19, 79, 101 and 639) (0.5 ml, 107 TCID50/ml) or PBS (3 animals) on the scarified skin of the right lower lip. (A). Clinical course of disease for OV-IA82-Δ119 (Δ119) and OV-IA82-RV119Flag (RV119). Results are shown for 5, 9, 16, and 21 days p.i. (B). Comparison of mean lesion length (in cm) at indicated time points. (TIF) [file ppat.1006779.s008.tif]
